# Pregnant alpha-1-microglobulin (A1M) knockout mice exhibit features of kidney and placental damage, hemodynamic changes and intrauterine growth restriction

**DOI:** 10.1038/s41598-020-77561-6

**Published:** 2020-11-26

**Authors:** Larysa Aleksenko, Bo Åkerström, Eva Hansson, Lena Erlandsson, Stefan R. Hansson

**Affiliations:** 1 Division of Obstetrics and Gynecology, Department of Clinical Sciences Lund, Lund University, Lund, Sweden; 2 Division of Infection Medicine, Department of Clinical Sciences Lund, Lund University, Lund, Sweden; 3 Obstetrics and Gynecology, Skåne University Hospital, Malmö, Lund, Sweden

**Keywords:** Medical research, Experimental models of disease

## Abstract

Alpha-1-microglobulin (A1M) is an antioxidant previously shown to be elevated in maternal blood during pregnancies complicated by preeclampsia and suggested to be important in the endogenous defense against oxidative stress. A knockout mouse model of A1M (A1Mko) was used in the present study to assess the importance of A1M during pregnancy in relation to the kidney, heart and placenta function. Systolic blood pressure (SBP) and heart rate (HR) were determined before and throughout gestation. The morphology of the organs was assessed by both light and electron microscopy. Gene expression profiles relating to vascular tone and oxidative stress were analyzed using RT-qPCR with validation of selected gene expression relating to vascular tone and oxidative stress response. Pregnant age-matched wild type mice were used as controls. In the A1Mko mice there was a significantly higher SBP before pregnancy that during pregnancy was significantly reduced compared to the control. In addition, the HR was higher both before and during pregnancy compared to the controls. Renal morphological abnormalities were more frequent in the A1Mko mice, and the gene expression profiles in the kidney and the heart showed downregulation of transcripts associated with vasodilation. Simultaneously, an upregulation of vasoconstrictors, blood pressure regulators, and genes for osmotic stress response, ion transport and reactive oxygen species (ROS) metabolism occurred. Fetal weight was lower in the A1Mko mice at E17.5. The vessels in the labyrinth zone of the placentas and the endoplasmic reticulum in the spongiotrophoblasts were collapsed. The gene profiles in the placenta showed downregulation of antioxidants, ROS metabolism and oxidative stress response genes. In conclusion, intact A1M expression is necessary for the maintenance of normal kidney, heart as well as placental structure and function for a normal pregnancy adaptation.

## Introduction

Alpha-1-microglobulin (A1M) is a low molecular weight protective protein that is conserved among vertebrates^1^. The main source of the protein in circulation is the liver, although it is synthesized in all cells and organs including the kidney, the skin and in the placenta during pregnancy^2–4^. Circulating plasma A1M is filtered through the glomeruli and rapidly taken up and metabolized in the proximal tubules. Alpha-1-microglobulin has several enzymatic properties that contribute to its physiological role as a protective antioxidant, i.e. reductase activity, and scavenging of heme and free radicals^5–7^. In addition, A1M is taken up by most cells and incorporated into the mitochondria by binding to Complex I of the respiratory chain^8^. Alpha-1-microglobulin has been shown to inhibit or reduce the damage caused by heme- and ROS-induced oxidative stress in several cell, organs, and animal models^9–15^. The gene expression of A1M is reported to be augmented by increased levels of ROS, heme and hemoglobin in the circulation^16,17^.

Preeclampsia (PE) and intrauterine growth restriction (IUGR) are severe pregnancy-related diseases that affect maternal and perinatal morbidity and mortality worldwide^18,19^. The etiology and pathogenesis are still not fully understood, but it is well established that both conditions are partially caused by a dysfunctional placenta, resulting in maternal endothelial damage and in severe cases also IUGR^20,21^. In PE, the maternal manifestations include hypertension and general organ damage^22^. Preeclampsia is associated with an elevated oxidative stress load^23^ and it has been shown that maternal plasma A1M levels are higher in PE pregnancies with or without IUGR compared to healthy pregnant women^24,25^. By using recombinant A1M as a pharmacological treatment in the ex vivo human placental perfusion model^13^ and in different animal PE models^11,12,26^, we showed that A1M protects and restores tissue and organ function, particularly in the placenta and kidney. This suggests that increased levels of A1M play an important role in the endogenous defense against the oxidative stress seen in PE. However, the upregulation is not enough to protect the tissues and organs from the damage resulting in the clinical manifestations of PE.

Recently we established an A1M knockout mouse model (A1Mko)^27^ to enable studies on the role of A1M in the normal non-pregnant physiology. These mice displayed hepatic ER stress, decreased bikunin levels in the circulation, a tendency toward increased organ weight and an overall age-dependent obesity compared to WT.

The hemodynamic changes during pregnancy present a unique challenge to the cardiovascular system as the increased blood volume leads to expansion of the peripheral vascular network and increased systemic vasodilation^28^. The cardiovascular system normally adapts to the progressively expanding peripheral vascular network and increased blood volume by increasing the heart rate, peripheral vasodilation, stroke volume and by compensatory cardiac hypertrophy^29,30^. The blood pressure homeostasis reflects how well the adaptive mechanisms are functioning.

In the present study we extended our investigation of the physiological role of A1M by examining kidney, heart and placenta function in pregnant A1Mko mice.

## Results

### Systolic blood pressure and heart rate

**Figure 1:**
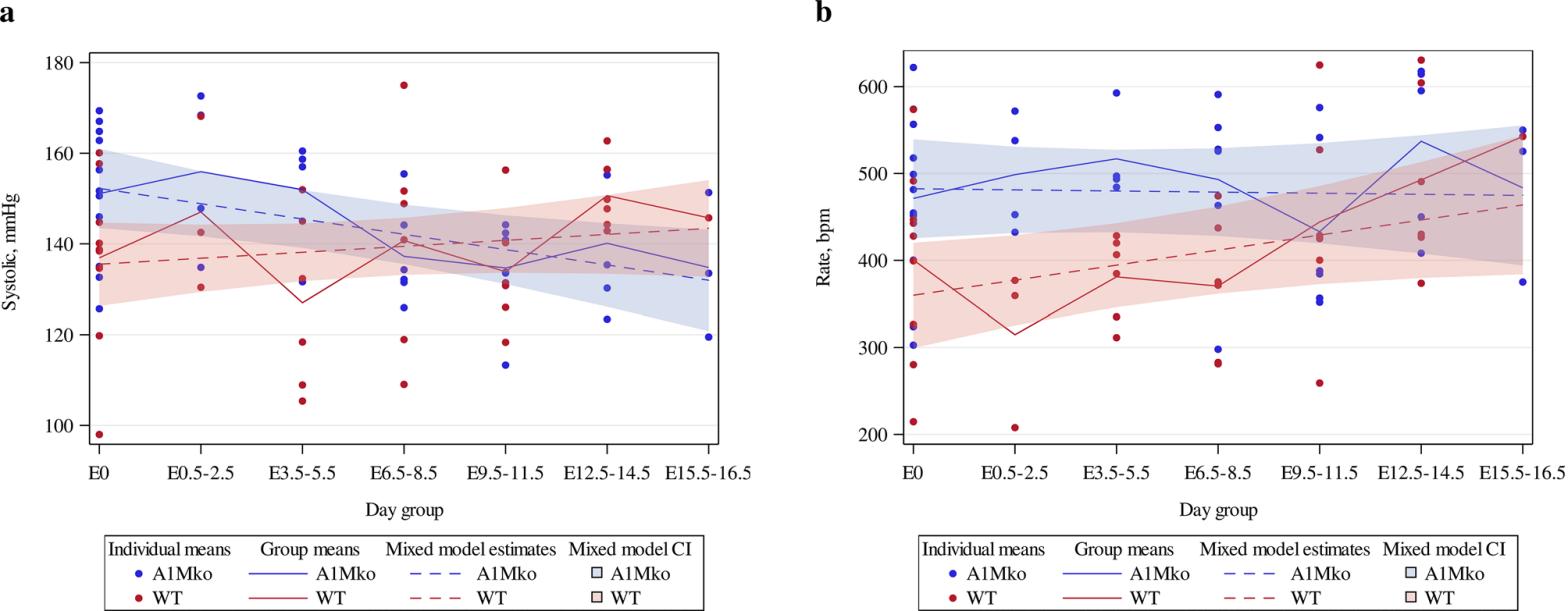
Trends in SBP (**a**) and HR (**b**) in the A1Mko and the WT mice before and during gestation analyzed with mixed regression modeling. (**a**) The baseline SBP was significantly higher (p = 0.0105) combined with a significant inverse association between SPB and gestational age (p = 0.0109) during pregnancy in the A1Mko when compared to the WT. (**b**) There was a significantly higher (p = 0.0047) baseline HR in the A1Mko mice when compared to the WT. HR did not change significantly during gestation in the A1Mko mice (p = 0.878), in contrast to the WT where there was a positive association between HR and gestational age (p = 0.0524).

The SBP was significantly higher in the non-pregnant A1Mko females compared to the WT (baseline difference 16.7 mmHg, 95% CI 4.0, 29.4, p = 0.0105, mixed regression model) (Table 1). The SBP trend in the A1Mko during pregnancy was different from that observed in the WT (Fig. 1a), with a significant inverse association between the SBP and gestational age in the A1Mko (slope − 3.4, 95% CI − 6.0, − 0.8, p = 0.0109, mixed regression model), while no association was seen in the WT (slope 1.3, 95% CI − 1.3, 4.0, p = 0.3221, mixed regression model). The slope difference between A1Mko and WT through gestation was statistically significant (slope difference − 4.7, 95% CI − 8.4, − 1.0, p = 0.0135, mixed regression model).

**Table 1:** Mixed regression model referencing for SBP and HR in A1Mko and WT before and during pregnancy.

Variable	Value estimated	Estimate (95% confidence interval)	P-value
SBP (mmHg)	Baseline A1Mko	152.2 (143.5; 161.0)	< 0.0001
	Baseline WT	135.5 (126.3; 144.7)	< 0.0001
	Baseline difference	16.7 (4.0; 29.4)	0.0105
	Slope A1Mko	− 3.4 (− 6.0; − 0.8)	0.0109
	Slope WT	1.3 (− 1.3; 4.0)	0.3221
	Slope difference	− 4.7 (− 8.4; − 1.0)	0.0135
HR (bpm)	Baseline A1Mko	482.4 (425.6; 539.3)	< 0.0001
	Baseline WT	359.8 (299.5; 420.2)	< 0.0001
	Baseline difference	122.6 (39.4; 205.8)	0.0047
	Slope A1Mko	− 1.3 (− 18.0; 15.4)	0.8775
	Slope WT	17.3 (− 0.2; 34.8)	0.0524
	Slope difference	− 18.6 (− 43.0; 5.8)	0.1310

Similarly, the HR was significantly higher in non-pregnant A1Mko compared to WT females (baseline difference 122.6 bpm, 95% CI: 39.4, 205.8, p = 0.0047, mixed regression model). The HR did not change significantly in the A1Mko during pregnancy (slope − 1.3, 95% CI − 18.0, 15.4, p = 0.8775, mixed regression model) (Table 1, Fig. 1b), while there was a weak positive association between the HR and gestational age in the WT mice (slope 17.3, 95% CI − 0.2, 34.8, p = 0.0524). The difference in slopes between A1Mko and WT during gestation was not significant (slope difference − 18.6, 95% CI − 43.0, 5.8, p = 0.1310, mixed regression model).

### Changes in tissue structure and function

#### Kidney

There was a significant increase in kidney weight (p = 0.037, Mann–Whitney) at E17.5, but not before pregnancy or at E12.5–13.5 in the A1Mko mice compared to the WT (Table 2).

**Table 2:** Morphological parameters in the A1Mko and the WT females at non-pregnant, E12.5-E13.5, and E17.5.

Parameters	A1Mko			WT		
	Non-pregnant (n = 3)	E12.5–E13.5 (n = 4)	E17.5 (n = 6)	Non-pregnant (n = 4)	E12.5–E13.5 (n = 4)	E17.5 (n = 6)
Kidneys (mg)	313 (291–360)	329 (289–394)	341^a^ (255–445)	280 (198–320)	282 (228–341)	279 (252–285)
Glomerular surface area (mm^2^)	3.2 (3.0–3.6)	3.5 (3.0–4.1)	3.2^b^ (2.9–3.6)	4.0 (3.1–4.3)	3.7 (3.5–4.6)	4.4 (4.1–5.8)
Glomerular cell count	47 (42–50)	50 (41–55)	42^c^ (34–52)	50 (49–60)	46 (41–52)	66 (53–71)
Heart (mg)	134^d^ (125–162)	143 (113–187)	124 (104–153)	99 (91–115)	111 (93–142)	117 (92–139)
Litter size	–	7 (3–8)	6 (4–10)	–	7 (6–7)	7 (7–9)
Labyrinth zone (µm)	–	68 (60–89)	68 (55–94)	–	52 (44–72)	73 (55–84)
Junctional zone (µm)	–	27 (14–30)	30 (23–42)	–	28 (24–34)	23 (15–42)
Fetal/placental weight ratio	–	2 (1–3)	6^e^ (4–8)	–	2 (1–2)	8 (7–14)

Data is presented as median (minimum − maximum).

Mann–Whitney test: ^a^P = 0.037, ^b,c^ P = 0.003, ^d^P = 0.034, ^e^P = 0.045.

**Figure 2:**
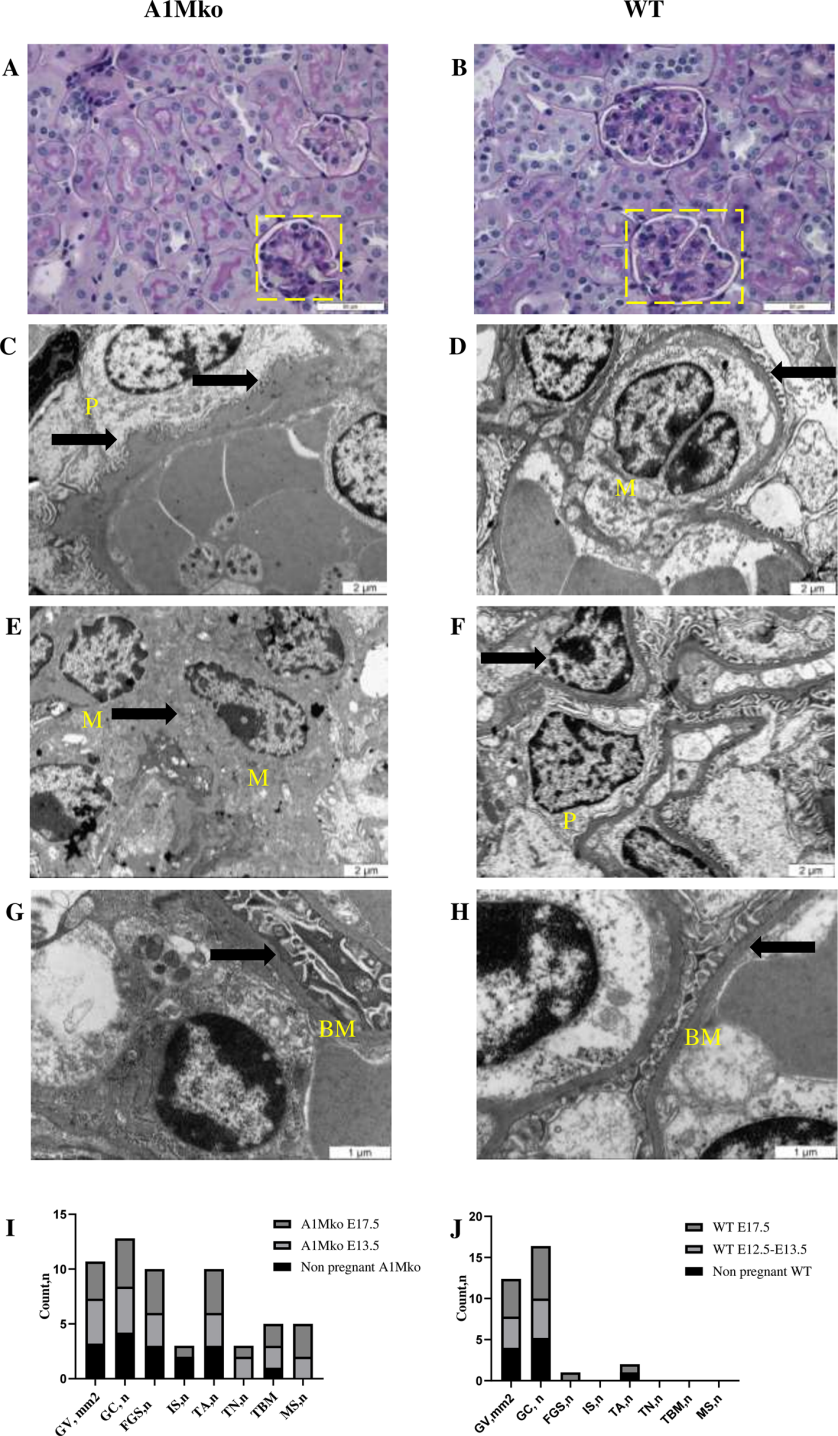
Microscopic analysis of the kidney: light microscopy (**a**,**b**), TEM (**c–h**) and summary of morphological abnormalities (**i,j**). (**a**) Glomerulus in the A1Mko at E17.5, PAS, 40X. There was a decrease in glomerular surface area (p = 0.003, Mann–Whitney) and glomerular cellularity (p = 0.003, Mann–Whitney) in the A1Mko compared to the WT at E17.5. (**b**) Glomerulus in the WT at E17.5, PAS, 40X. (**c**) Thickened basement membrane in the A1Mko at E13.5 (arrows), bar 2 µm. (**d**) Normal basement membrane in the WT (arrow) at E13.5, bar 2 µm. (**e**) Mesangial widening and sclerosis in the A1Mko at E17.5 (arrow), bar 2 µm. (**f**) Normal renal mesangium in the WT at E17.5 (arrow), bar 2 µm. (**g**) Fused foot processes in the A1Mko at E17.5 (arrow), bar 1 µm. (**h**) Normal foot processes in the WT at E17.5 (arrow), bar 1 µm. (**i**) Summary of renal light microscopy (n = 13) and ultrastructural (n = 7) morphological features in the A1Mko before and during pregnancy. Total count of positive cases. Focal glomerular sclerosis (p = 0.007, Fisher exact test) and tubular atrophy (p = 0.026, Fisher exact test) were more common in both non-pregnant and pregnant A1Mko than in the WT. Interstitial scarring and tubular necrosis were only detected in the A1Mko (Table 2). Basement membrane thickening, mesangial expansion with sclerosis and fusion of foot processes were observed by TEM, to some degree already before pregnancy and more during it in the A1Mko, but not in the WT. (**j**) Summary of renal light (n = 14) and ultrastructural (n = 6) morphologic features found in the WT before and during pregnancy. Total positive case count. *BM* basement membrane, *FGS* focal glomerular sclerosis, *FPF* fused foot processes, *GC* number of cell per glomerulus, *GSA* glomerular surface area, *IS* interstitial sclerosis, *M* mesangium, *MS* mesangial sclerosis, *P* podocyte, *TA* tubular atrophy, *TBM* thickening of basement membrane, *TN* tubular necrosis.

A statistically significant decrease in the glomerular surface area (p = 0.003, Mann–Whitney) and glomerular cellularity (p = 0.003, Mann–Whitney) was seen in the kidneys of the pregnant A1Mko females at E17.5 when compared to the controls (Table 2, Fig. 2**a,b)**, but not before pregnancy or at E12.5–13.5.

Increased renal abnormalities were seen by light microscopy and TEM in the A1Mko mice compared to the WT at all three time points (Table 3, Fig. 2**i,j**). Focal glomerular sclerosis (p = 0.007, Fisher exact test) and tubular atrophy (p = 0.026, Fisher exact test) were more common in both non-pregnant and pregnant A1Mko than in the WT. Interstitial scarring and tubular necrosis were only detected in the A1Mko (Table 3). Basement membrane thickening (Fig. 2c,d), mesangial expansion with sclerosis (Fig. 2**e,f**) and fusion of foot processes (Fig. 2**g,h**) were observed by TEM, to some degree before and more during pregnancy in the A1Mko, but not in the WT.

**Table 3:** Light microscopy analysis and ultrastructural analysis by TEM in the A1Mko and the WT at non-pregnant, E12.5-E13.5 and E17.5.

Light microscopy features, number of cases*(n)	A1Mko			WT		
	Non-pregnant (n = 3)	E12.5–E13.5 (n = 4)	E17.5 (n = 6)	Non-pregnant (n = 4)	E12.5–E13.5 (n = 4)	E17.5 (n = 6)
Focal glomerular sclerosis	3	3	4	0	0	1
Interstitial scarring	2	0	1	0	0	0
Tubular atrophy	3	3	4	1	0	1
Tubular necrosis	0	2	1	0	0	0
Total	8	8	10	1	0	2

Ultrastructural changes	Non-pregnant (n = 2)	E12.5-E13.5 (n = 2)	E17.5 (n = 3)	Non-pregnant (n = 2)	E12.5-E13.5 (n = 2)	E17.5 (n = 2)
Thickening of basement membrane,	1	2	2	0	0	0
mesangial widening and sclerosis	0	2	3	0	0	0
Fused foot processes	1	2	3	0	0	1
Total	2	6	8	0	0	1

*(n) = Positive cases counted. At least two sections per examined kidney were used for light microscopy and two randomly selected glomeruli per kidney for TEM.

#### Heart

**Figure 3:**
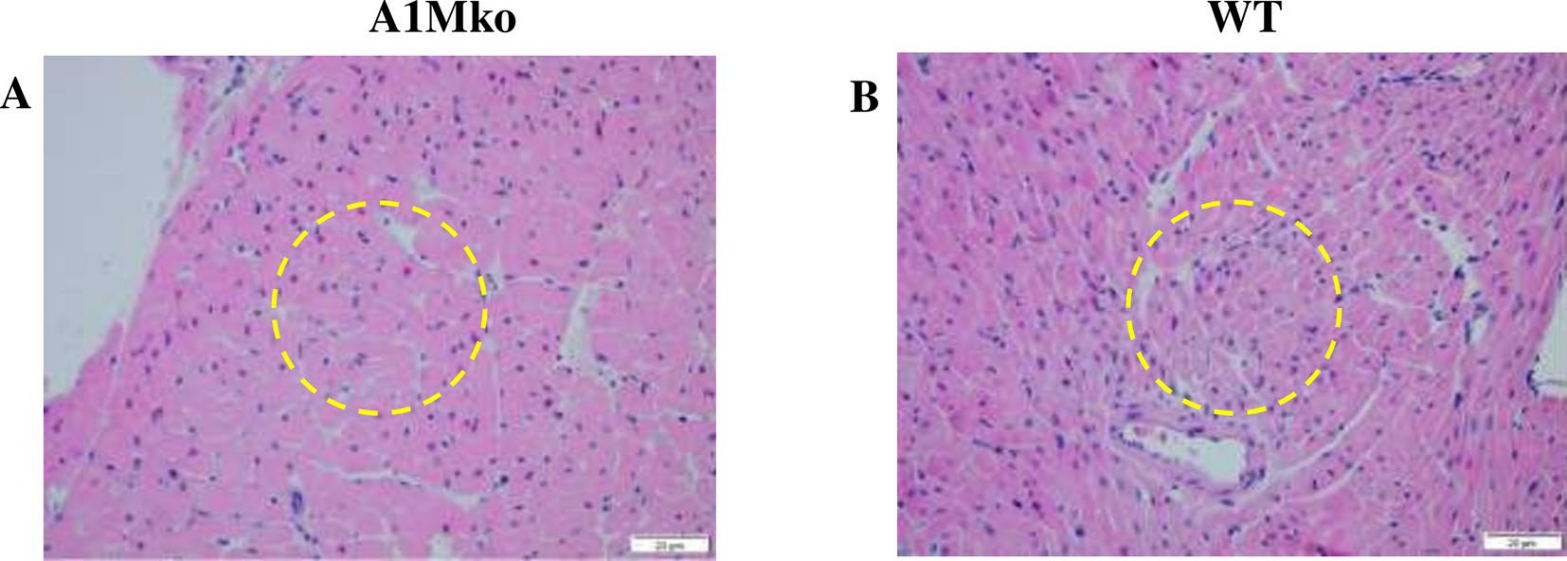
Light microscopy analysis of the heart in the A1Mko and WT mice. (**a**) Left ventricle with features of cardiac hypertrophy in the A1Mko, E 17.5, H&E, 20X. There is an increased diameter of the individual cardiac myocytes on a transverse cut section. The encircled area highlights the cellular changes. (**b**) Normal myocardium in the WT, E17.5, H&E, 20X. Cardiac myocytes consist of population of cells with similar diameter on a transverse cut section.

There was significantly higher heart weight in the A1Mko before pregnancy compared to the WT (p = 0.034, Mann–Whitney), but no significant differences in heart weights between the A1Mko and the WT females were noted during pregnancy (Table 2). Light microscopy of the heart showed cellular hypertrophy in the A1Mko before and during pregnancy, but this difference was not significant (Table S1). There was no evidence of necrosis or scarring in the myocardium (Fig. 3).

#### Placentas and fetuses

**Figure 4:**
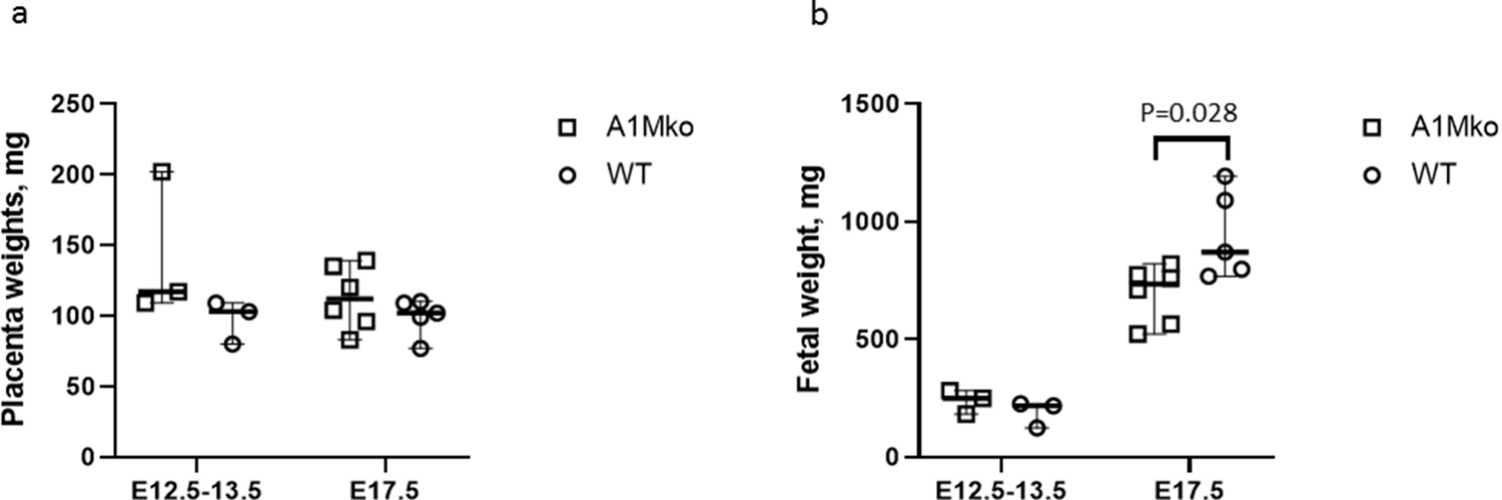
Means per litter (squares for the A1Mko, circles for the WT), median (thick horizontal lines) and range (thin vertical lines) for placenta and fetal weights in A1Mko (n = 3) and WT (n = 3) mice at E12.5- 13.5 and A1Mko (n = 6) and WT (n = 5) mice at E17.5. (**a**) There were no statistical differences in placental weight between A1Mko and WT at the two gestational points. (**b**) The fetuses were significantly lighter in the A1Mko compared to the WT at E 17.5 (p = 0.028, Mann–Whitney), but not at E12.5–13.5.

There were no differences in litter sizes at the mid and late gestation between the A1Mko and the WT (Table 2). There was no significant difference in placental weights at E12.5–13.5 and E17.5 between A1Mko and WT (Fig. 4a). The fetal weight in the A1Mko was significantly lower (p = 0.028, Mann–Whitney) when compared to the WT at E17.5, but not at E12.5–13.5 (Fig. 4b).

The fetal/placental weight ratio was significantly lower in the A1Mko (p = 0.045, Mann–Whitney) compared to the WT at E17.5 (Table 2).

There was no significant difference in the width of labyrinth zone and junctional zone in the placentas of the A1Mko compared to the WT at E12.5–13.5 and E17.5 (Table 2).

**Figure 5:**
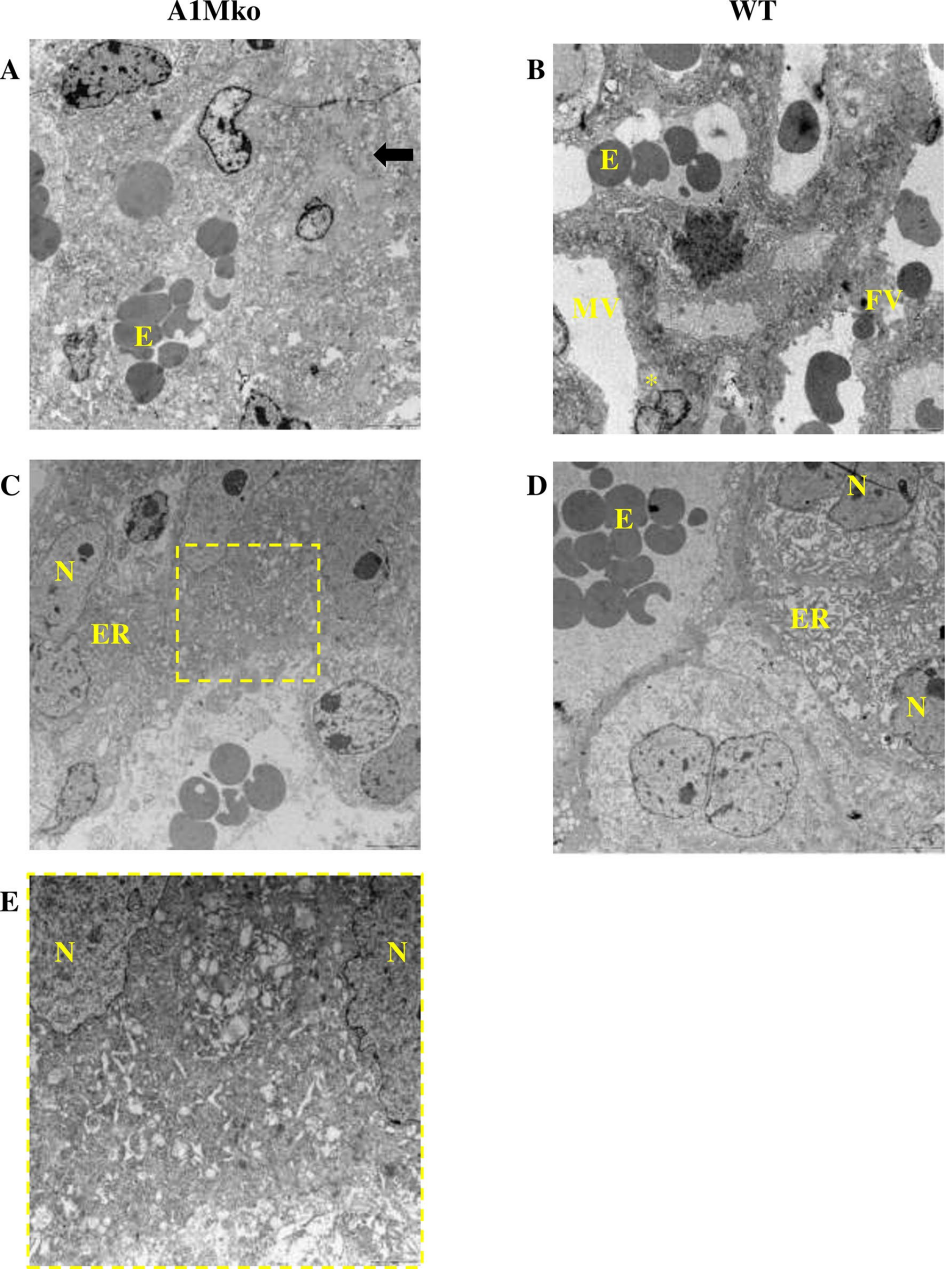
TEM analysis of the placentas. (**a**) Collapsed vessels of the labyrinth in the A1Mko at E17.5**,** bar 5 µm. Normal maternal and fetal compartments are effaced and replaced with debris. Some remnants of syncytiotrophoblatsts are present (arrow). (**b**) Normal vessels in the labyrinth of the WT at E17.5, bar 5 µm. There is a clear separation of maternal and fetal vascular compartments. The star indicates the cytotrophoblast in the maternal vessel. (**c**) Collapsed ER (arrow) in the spongiotrophoblast in the A1Mko at E 17.5, bar 2 µm. The characteristic dilated cysternae of ER (lace-like pattern) is not visible. Selected area is magnified in (**e**). (**d**) Normal ER in spongiotrophoblast in the WT at E 17.5, bar 2 µm. *E* erythroblast, *ER* endoplasmic reticulum, *FV* fetal vessel, *MV* maternal vessel, *N* nucleus, *FE* fetal endothelium, *MV* maternal vessel, *ST* spongiotrophoblast.

No morphological abnormalities were noted in the placenta in the A1Mko compared to the WT by light microscopy, but TEM revealed areas of vascular collapse in the labyrinth zone (Fig. 5**a,b**) and collapsed rough endoplasmic reticulum (ER) in the spongiotrophoblasts (Fig. 5**c–e**) in the A1Mko at E17.5.

### Urine and blood biochemistry

Urinary albumin and creatinine excretion in the A1Mko at baseline and up to E17.5 were not significantly different compared to WT animals (Table S2). Similarly, no significant difference was found in blood urea nitrogen (BUN) between the A1Mko and the WT either before or during pregnancy (Table S2).

### Gene profiling

Selected gene expression in the kidney, heart and placenta during pregnancy was evaluated in the A1Mko and the WT females by using two different RT^2^ profiler PCR arrays: Mouse Hypertension and Mouse Oxidative stress and Antioxidant Defense.

The genes were allocated to groups according to function as shown in Table 4. These were: vasodilation, vasoconstriction, blood pressure regulation, osmotic stress response, ion transport, ROS metabolism and oxidative stress response. See Table S3 for the complete list of results for all genes present on the RT^2^ PCR profiler arrays.

**Table 4:** Significant fold changes in gene expression in the A1Mko at E12.5–13.5 and E17.5 compared to the WT.

Gene symbol	Gene	Fold change X	Gestational age
**Kidneys**			
Vasodilation			
*NPPB*	Natriuretic peptide type B	+ 13.16	12.5–13.5
*KNG1*	Kininogen 1	− 2.01	17.5
*CPS1*	Carbamoyl-phosphate synthetase 1	− 2.39	17.5
*AGTR2*	Angiotensin II receptor type 2	− 3.03	17.5
*MB*	Myoglobin	− 76.54	17.5
Vascular constriction			
*MYLK2*	Myosin, light polypeptide kinase 2	+ 4.22	17.5
*EDN2*	Endothelin 2	+ 2.71	17.5
*ANDRA1D*	Adrenergic receptor alpha 1d	+ 2.67	17.5
*UTS2R*	Urothensin 2 receptor	+ 2.59	17.5
*PTGIR*	Prostaglandin I receptor (IP)	+ 2.25	17.5
*UTS2*	Urotensin 2	+ 2–21	17.5
*PTGS2*	Prostaglandin-endoperoxide synthase2	+ 2.07	17.5
*DRD3*	Dopamine receptor D3	− 2.12	12.5–13.5
Blood pressure regulation and osmotic stress response			
*AVPR1B*	Arginine vasopressin receptor 1b	+ 3.35	17.5
*BDKRB2*	Bradykinin receptor beta 2	+ 2.21	17.5
Ion transport			
*CNGA2*	Cyclic nucleotide gated channel alpha2	+ 3.12	17.5
*CNGA3*	Cyclic nucleotide gated channel alpha3	+ 2.45	17.5
*CNGB1*	Cyclic nucleotide gated channel beta1	+ 2.17	17.5
*CNGA1*	Cyclic nucleotide gated channel alpha1	+ 2.07	17.5
ROS metabolism			
*NOXA1*	NADH oxidase activator 1	+ 2.66	17.5
*NOS2*	Nitric oxide synthase inducible	+ 2.44	17.5
*GPX5*	Glutathione peroxidase 5	+ 2.13	17.5
Oxidative stress response			
*AOX1*	Aldehyde oxidase	− 2.10	17.5
**Heart**			
Vasodilation			
*AGTR2*	Angiotensin II receptor type 2	+ 2.59	17.5
*CPS1*	Carbamoyl-phosphate synthetase 1	− 8.81	17.5
*KNG1*	Kininogen 1	− 18.94	17.5
Vascular constriction			
MYLK2	Myosin, light polypeptide kinase 2	+ 2.04	17.5
**Placenta**			
Antioxidants			
*TXNRD2*	Thioredoxin reductase 2	+ 2.21	12.5–13.5
*GPX5*	Glutathione peroxidase 5	− 2.43	17.5
*GPX2*	Glutathione peroxidase 2	− 2.94	12.5–13.5
*EHD2*	EH-domain containing 2	− 3.07	12.5–13.5
*MPO*	Myeloperoxidase	− 4.12	12.5–13.5
ROS metabolism			
*AOX1*	Aldehyde oxidase	− 2.15	17.5
*NOXA1*	NADH oxidase activator 1	− 2.27	17.5
*FMO2*	Flavin containing monooxygenase2	− 3.29	12.5–13.5
Oxidative stress response			
*CCL5*	Chemokine (C–C motif) ligand5	− 2.31	12.5–13.5
*APOE*	Apolipoprotein E	− 3.17	12.5–13.5
*KRT1*	Keratin 1	− 3.26	12.5–13.5
*KRT1*	Keratin 1	− 4.64	17.5

X Fold change represents upregulated (+) or downregulated (−) gene expression. The threshold of two-fold up- or downregulation of expression is statistically significant (Student’s T-test, p < 0.05) in the A1Mko compared to the WT after normalization against housekeeping genes.

### General gene expression patterns

**Figure 6:**
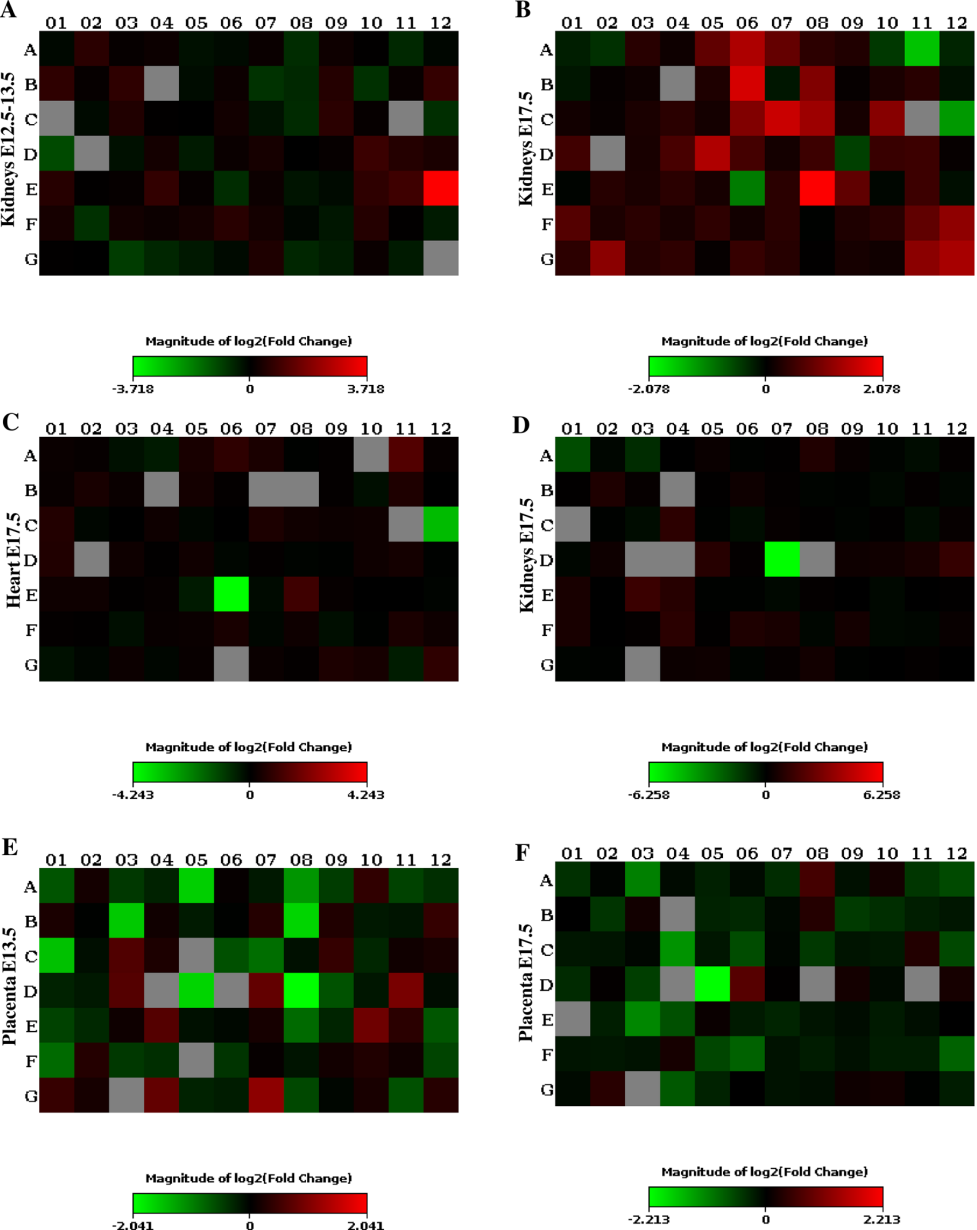
Full gene expression profiles in the A1Mko relative to WT. (**a**) Hypertension array, kidneys at E 12.5–13.5. (**b**) Hypertension array, kidneys at E 17.5. (**c**) Hypertension array, hearts at E 17.5. (**d**) Oxidative Stress and Antioxidant Response array, kidneys at 17.5. (**e**) Oxidative Stress and Antioxidant Response array, placentas at E12.5–13.5. (**f**) Oxidative Stress and Antioxidant Response array, placentas at E 17.5. The images represent the full gene expression profile presented in Table S3. The color spectrum for each image shows the range of fold changes observed in the array from downregulation (green) to upregulation (red) based on the threshold cycle (C_T_) above the base line and housekeeping genes (ΔΔ (delta delta) C_T_ method). The gray color means no expression. Each square represents a specific gene.

A general upregulation of A1Mko kidney genes compared to WT genes in the hypertension array was seen at both time points (Fig. 6**a,b**). These were mostly genes involved in vasoconstriction, blood pressure regulation, osmotic stress response and ion transport. The most significantly downregulated genes in the hypertension array in the A1Mko at E17.5 were vasodilators, in both the kidney and heart (Fig. 6**b,c**). In contrast, most genes of the Oxidative Stress Response and Antioxidant Response array were downregulated in the A1Mko kidney and placenta (Fig. 6**d–f**). These belonged mostly to the antioxidant, ROS metabolism and oxidative stress response categories.

### Specific genes of the kidneys

Table 4 shows a list of all significantly up- or downregulated genes, categorized in functional groups, in the A1Mko organs relative to WT. In the kidneys at E12.5–13.5, the natriuretic peptide type B *(NPPB*) a vasodilator and a marker for cardiac overload, was upregulated (+ 13.16X). In contrast, at E17.5 there were several vasodilators that were downregulated (Table 4, Fig. 6**a,b**), i.e. myoglobin (*MB*, − 76.5X), angiotensin II receptor type 2 (*AGTR2),* carbamoyl-phosphate synthetase 1(*CPS1)* and kininogen 1 *(KNG1).*

One vasoconstrictor was downregulated in the kidney at E12.5–13.5, dopamine receptor D3 (*DRD3*), while at E17.5 all other vasoconstrictor genes were upregulated, with myosin light polypeptide kinase 2 *(MYLK2)* being the highest (+ 4.22). Additionally, genes involved in blood pressure regulation and osmotic stress response (e.g. Arginine vasopressin receptor 1b (*AVPR1B*)), ion transport (Cyclic nucleotide gated channel genes (*CNG*s)) and ROS metabolism were upregulated in the A1Mko kidneys, while one oxidative stress response gene, aldehyde oxidase (*AOX1*), was downregulated (Table 4).

### Specific genes of the heart

The changes in gene expression in the heart were similar to those observed in the kidneys, the vasodilators being downregulated at E17.5. These included *KNG1* and *CPS1* (Table 4, Fig. 6**c**). However, the vasodilator *AGTR2* and the vasoconstrictor *MYLK2* were upregulated at E17.5.

### Specific genes of the placenta

At E12.5–13.5, several genes allocated to the antioxidant category were downregulated in the A1Mko placentas. These were myeloperoxidase (*MPO*), EH-domain containing 2 (*EHD2*), and glutathione peroxidases 2 (*GPX*2) (Table 4, Fig. 6**e,f**). In addition, there was a downregulation of the ROS metabolism gene flavin containing monooxygenase 2 (*FMO2*) and the oxidative stress response genes keratin 1 *(KRT1)*, apolipoprotein E (*APOE)* and chemokine (C–C motif) ligand 5 (*CCL5)*. The only gene that was upregulated at E12.5–13.5 was the antioxidant thioredoxin reductase 2 (*TXNRD2*). At E17.5, glutathione peroxidase 5 (*GPX5*), two ROS metabolism genes and the oxidative stress response gene *KRT1* were downregulated, while no genes were upregulated.

### Gene profiles validation

Selective validation of genes with individual probes confirmed downregulation of *Mb* (p = 0.016, Mann–Whitney) and upregulation of the vasodilator gene *MYLK2* (p = 0.032, Mann–Whitney) in the kidneys at E17.5 (Table 5, Fig. S1**c,e**).

**Table 5:** Validation RT-qPCR demonstrating fold changes in gene expression in the A1Mko at E12.5–13.5 and E17.5 compared to the WT.

Gene symbol	Gene	Fold change	Gestational age	p-value
**Kidneys**				
Vasodilation				
*NPPB*	Natriuretic peptide type B	+ 13.31	12.5–13.5	0.200
*CPS1*	Carbamoyl-phosphate synthetase 1	− 4.5	17.5	0.222
*MB*	Myoglobin	− 6.3	17.5	0.016
*KNG1*	Kininogen 1	− 2.4	17.5	0.151
Vascular constriction				
*MYLK2*	Myosin, light polypeptide kinase 2	+ 1.72	17.5	0.032
**Heart**				
Vasodilation				
*CPS1*	Carbamoyl-phosphate synthetase 1	− 3.0	17.5	0.151
Vascular constriction				
*MYLK2*	Myosin, light polypeptide kinase 2	+ 1.42	17.5	0.421
**Placenta**				
Oxidative stress response				
*APOE*	Apolipoprotein E	− 2.5	12.5–13.5	0.400

X Fold change represents upregulated (+) or downregulated (−) gene expression in the validation RT-qPCR for each gene in the A1Mko compared to the WT after normalization against one housekeeping gene. The p-value was calculated using Mann–Whitney test.

However with individual probes, upregulation of *NPPB* gene at E12.5–13.5 and downregulation of *CPS1* and *KNG1*at E17.5 in the kidney were not confirmed (p = 0.200, p = 0.222 and p = 0,151 respectively, Mann–Whitney) (Table 5, Fig. S1**a,b,d**).

Change in *CPS1* and *MYLK2* genes in the heart at E17.5 were also not confirmed (p = 0.151 and p = 0.421 respectively, Mann–Whitney) (Table 5, Fig. S1**f**,**g**), as was downregulation of *APOE* in the placenta at E12.5–13.5 (p = 0.400, Mann–Whitney) (Table 5, Fig. S1**h**).

## Discussion

In this work, the A1Mko mouse line was employed to investigate the role of the reductase, heme- and radical-binding protein A1M in pregnancy. To evaluate the impact of A1M-loss during pregnancy, the biology of A1Mko female mice was first considered. It was previously shown that the non-pregnant A1Mko phenotype is characterized by ER stress and increased antioxidant expression in the liver, as well as obesity and increased weight of individual organs^27^. In this study we continued to characterize the A1Mko phenotype and showed that non-pregnant A1Mko females have significantly increased SBP and HR as well as a tendency toward increased heart weight. Thus, there was an indication of a compromised cardiovascular system in the non-pregnant A1Mko phenotype, which was also clearly detected in early and late pregnancy. Likewise, the increased kidney weight and the kidney tissue morphology of the non-pregnant females suggest that an abnormal kidney function was present even before pregnancy. All together, these results indicate an altered cardiovascular and kidney function in both non-pregnant and pregnant A1Mko females compared to WT females.

During pregnancy, the A1Mko female mice showed signs of deranged expression of vaso-regulatory genes, structural abnormalities in the kidney and the placenta, as well as compromised fetal growth. The major physiological events of pregnancy, placental development and fetal growth showed several signs of dysregulation in the A1Mko females. The placenta showed collapsed vessels in the labyrinth zone and ER stress in spongiotrophoblasts, which may impact the feto-placental exchange of gases and nutrients and result in IUGR. As expected, there was a reduced weight of the fetuses at term and a reduced fetal/placental weight ratio.

Early in a normal pregnancy, as the maternal blood volume increases, there is an increased vasodilation and decreased peripheral resistance leading to increased cardiac output^31^. Decreased vascular tone (vasodilation) or increased vascular tone (vasoconstriction) affect fluid volume, blood flow and oxygen transport^32,33^, as well as nutrient supply to the fetus. Vasodilation is reported to have a peak at mid gestation and to gradually decrease toward term as part of the normal baroreflex response in pregnancy^34^. However, our results suggest that this normal physiological response failed in the A1Mko.

The importance of A1M in kidney function was previously reported in male Wistar rats, where it was demonstrated that the protein can reduce fetal hemoglobin-induced glomerular permeability^14^. This protective effect has also been shown in a pregnant rabbit model^12^. Kidney damage is typically seen in the pregnancy-related complication, PE, and is thought to be due to a deranged mechanism for adapting to changes in fluid volume in pregnancy^35–37^. This is partially attributed to reduced levels of vascular endothelial growth factor and increased levels in the circulation of soluble fms-like tyrosine kinase one, leading to decreased vasodilation^38,39^. This is clinically seen as glomerular endotheliosis, a pathognomonic finding in PE^39^. In the present study, the histological findings were not the typical findings reported in PE^36,40^ except for the basement membrane thickening, fusion of foot processes and mesangial expansion^40,41^. However, the changes do indicate damage to the glomerular filtration barrier, which compromises renal function^40,42^. In general changes in oncotic pressure might significantly affect blood volume regulation and therefore the blood pressure in a normal physiological response^43^. These need to be considered in follow-up studies with the A1Mko model. In humans, A1M levels have been reported to increase during the course of normal pregnancy, but at a higher rate in PE^24,25^. The current findings suggest that the reported increase of A1M levels may be an adaptive mechanism to prevent kidney and placental damage.

The gene expression profiles indicate an imbalance in the maintenance of vascular tone, tending toward increased vasoconstriction. This includes increased expression of the cyclic nucleotide gated ion channel genes (CNGAs) and AVPR1B. The CNGAs are the only targets of cyclic guanosine 3′,5′-monophosphate (cGMP) for vascular smooth muscle contraction in the kidneys^44^. The cGMPs are generated from guanosine triphosphate in response to natriuretic peptides when there is increased pressure and stretch in the vasculature which accompany high volume, leading to vasoconstriction^45,46^. The AVPR1B is important for fluid homeostasis, cardiovascular function and blood pressure regulation during pregnancy^47,48^. In mice, low dose infusions of vasopressin have been shown to cause PE-like manifestations, including IUGR^49^.

The attenuated expression of *MB* is very interesting. Myoglobin is a potent inducer of vasodilation under hypoxic conditions. By reacting with nitrite^50^ to generate nitric oxide (NO) it causes vasodilatation. In rodents, NO inhibition during pregnancy leads to hypertension^51,52^. When the kidney tubules have a limited oxygen supply, due to shunting in the medulla, the induced hypoxic condition causes an increase in *MB* expression as a compensatory vasodilation mechanism^53^. The failed response seen in the A1Mko model suggests a weakening of the normal adaptive hemodynamic response. The reason for the downregulation of *MB* in the A1Mko kidneys is not known, but it may be speculated that A1M, as a heme-binding protein^54^, is involved in the regulation of cell-free heme levels. Since heme is a component of myoglobin, the lack of A1M may compromise the incorporation of heme groups into the MB molecule.

To the best of our knowledge, there is currently no reported data on mouse renal expression of the *NPPB* gene. The *NPPB* protein is a potent diuretic, natriuretic and vasodilator^55^ contributing to blood volume control by the heart and the kidneys^56^. Recently, the protein has been proposed as a marker of early onset PE^57,58^. It is interesting that the expression was seen only at E12.5–13.5 in the kidneys and was absent at E17.5, further suggesting a failed hemodynamic adaptation.

The major physiological defect associated with the A1Mko pregnancy model in the kidneys, heart and placenta is the vascular tone. Failure in regulating and maintaining vascular tone results in increased SBP and HR and the collapsed vessels seen in the placenta, contributing to IUGR. An antiangiogenic imbalance is a well described situation in pregnancies complicated by PE, with and without IUGR, hypertension, gestational diabetes and heart failure^59–63^. Models of pregnancy-induced hypertension such as the reduced utero-placental pressure and renin-angiotensin system in rats, demonstrate imbalance in vascular tone^63,64^.

The major limitation of this study is the small sample size. One reason for the small sample size, as stated previously, was the inherent stringency in the use of the CODA instrument for SBP and HR measurements based on the volume-pressure method validated in non-pregnant rodents. The mice needed to have the recommended normal temperature range at the time of the measurement, which is a critical test requirement for validation of the results. For some mice it was also stressful to fit into the restraining tube at the later stages of gestation, and therefore they were excluded from further SBP and HR measurements. As a result, the regression line for pregnant A1Mko after E8.5 and later in pregnancy has to be interpreted with caution. In addition, considering that the use of individual probes could not validate the results of some of the pooled samples, we recommend that some of the gene expression data should be interpreted with caution.

The animals used to assess the litter size differences were limited to comparable numbers obtained in the SBP and HR data. A follow-up study with large number of A1Mko mice is necessary to address the limitations of the present study.

## Conclusion

In conclusion, we report that loss of A1M is associated with abnormal cardiovascular function in non-pregnant mice and that A1M is important for cardio-vascular adaptation and the development of a functional placenta to support fetal growth.

## Material and methods

### Ethics committee approval

The study protocol was approved by the ethics committee for animal studies at Lund University, Sweden (permission M77-14, 2014). Animal experiments were carried out in strict adherence to the guidelines of the Code for Method and Welfare Considerations in Behavioral Research with Animals (Directive 86/609EC). All efforts were made to minimize suffering.

### Animals

The A1Mko line was established as previously described^27^. The model has a selective deletion of exon 2 to exon 6 in the alpha-1- microglobulin bikunin precursor gene, resulting in a complete loss of expression of the A1M protein.

All mice were kept at the Biomedical Center Animal Facility, Lund University, Sweden. They were maintained on 12 h light/dark cycle *ad libitum* on standard laboratory rodent chow and water. Only mice from 18 weeks of age were included in the study.

Ten A1Mko and ten WT females were employed for breeding of the A1Mko or WT lines, respectively. The gestational age was counted from the date of a visible copulation plug, defined as day E0.5. From these animals, four pregnant A1Mko and four pregnant WT mice were sacrificed at E12.5–13.5, while six pregnant A1Mko and six pregnant WT mice were sacrificed at E17.5. An additional three A1Mko and four WT non-pregnant females were sacrificed for baseline values. The age at termination was 25 ± 3 weeks for the A1Mko females and 24 ± 2 weeks for the WT females.

### Blood pressure and heart rate measurements

At 18 weeks of age, SBP and HR measurements were performed on the females for at least three weeks prior to breeding, which included one week of training. The SBP and HR were measured with a non-invasive tail cuff device (CODA8 with four channels, Kent Scientific, CT, USA), which uses a volume-pressure method. All measurements were performed on conscious restrained animals positioned on a heated platform following a standard protocol provided by the manufacturer. All measurements on mice in vivo were held between 10:00 and 11:00 a.m. During the training period mice were made accustomed to being handled by the researcher. This involved placing them in an animal restrainer of appropriate size that was placed on a heated platform, training them to tolerate the placing of a tail cuff and later the deflation/inflation cycle and the sound of the working equipment. Stress monitoring was carried out throughout the experiment by observing the mice while monitoring the heart rate and the body temperature. Mice were considered stressed and returned to the cage if they displayed increased body movements, excessive movements or retraction of the tail, rapidly increasing heart rate or a body temperature above 37.6 for > 3 subsequent measurements. Stressed mice were not included in the SPB and HR measurements again on that day^65–67^.

Trained mice were followed for two weeks to establish the baseline SBP and HR. After conception, daily measurements were taken until one day before termination. A session per day consisted of 10–15 min for thermo-regulation when restrained mice were positioned on the heated platform to achieve normal temperature, and of two sets of 15 measurements on each mouse which lasted for eight minutes and 40 s in total. Each set consisted of five acclimatization cycles and 10 true inflation/deflation cycles. Stress monitoring was carried out in a similar way to during the training period. All data obtained per day was checked and all measurements which did not pass the internal software control or were above/below the normal temperature range (34–37 °C) were excluded from the analysis^65–67^.

### Urine and blood samples

Urine and blood samples were collected at the time of termination. Briefly, following anesthesia with an isoflurane/oxygen mix, a middle line abdominal incision was made, and urine was collected trans-vesicularly with a sterile syringe. The urine was stored at − 80 °C until further analyses were conducted.

Blood was collected from the inferior vena cava into lithium-heparin treated vacuum tubes. Plasma was separated from whole blood by centrifugation at 2000 RPM for 10 min at room temperature and stored at − 80 °C until further analyses were conducted.

### Tissue harvesting and processing

Following anesthesia, blood was sampled, and all animals were euthanized by cervical dislocation. The kidney, heart, placentas and live fetuses were harvested and weighed. The number of dead fetuses was counted for each mouse.

#### Kidneys

Two cubes of tissue of 1–2 mm^3^ were taken from the cortex and medulla of one kidney and put into freshly made fixative consisting of 1.5% paraformaldehyde and 1.5% glutaraldehyde. The fixed tissues were processed for TEM as described^68^. The remaining kidney tissue was frozen on dry ice and kept at -80 °C until used for RNA extraction. The other whole kidney was put into 4% buffered formaldehyde and fixed for 24 h at room temperature. It was then paraffin embedded and cut into 5 µm sections to be used for light microscopy analysis using hematoxylin and eosin (H&E), Periodic acid Schiff (PAS) and Masson Trichrome staining techniques.

#### Heart

Each heart was sectioned along the septum, dividing it into the right and left compartments. The left compartment was processed for light microscopy and the right was frozen on dry ice and later used for RNA extractions.

#### Placenta

Five randomly selected placentas were harvested from each pregnant female and used as follows: two placentas were prepared for light microscopy analysis, one placenta was prepared for TEM analysis and two whole placentas were frozen on dry ice and used for RNA extraction.

### RNA extraction

RNA was purified using a TRIzol-chloroform-ethanol method and the RNeasy mini kit (Qiagen, TX, USA). In brief, the tissue was homogenized in TRIzol reagent (Invitrogen, CA, USA) and centrifuged. The supernatant was mixed with chloroform, centrifuged again and the supernatant collected and mixed with an equal amount of ethanol. The mixture was put on a RNeasy mini spin column and RNA was purified in accordance with the protocol supplied by the manufacturer. The RNA sample quality was assessed by using the Agilent Bioanalyzer 2100 system (Agilent Technologies, CA, USA). The RNA integrity number for samples selected for further analysis was 9.1 ± 0.6.

### Tissue morphology

#### Kidneys

Histological examination, photographic documentation, measurements and volume calculations were performed using 20X and 40X magnifications with an Olympus microscope (Olympus, Tokyo, Japan) and the Life Science Imaging Olympus Software (Olympus, Tokyo, Japan). The renal tissue evaluation was performed on two serial sections per specimen in three non-pregnant A1Mko, four pregnant A1Mko at E12.5–13.5 and six pregnant A1Mko at E17.5, four non-pregnant WT, four pregnant WT at E12.5–13.5 and six pregnant WT at E17.5.

Ten cortical glomeruli, sectioned close to the midline with the vascular pole and the whole circumference of the Bowman’s capsule visible, were examined and measured on PAS staining at 40X magnification. Glomeruli with evidence of fibrosis by PAS or sectioned tangentially were excluded from the measurements. A cell count was performed on the same glomeruli photographed at 40X magnification.

Analysis by TEM was performed on the kidney from two A1Mko and two WT females at E12.5–13.5 and three A1Mko and two WT females at E17.5. Additionally, two non-pregnant A1Mko and two WT females were examined for baseline values. At least two randomly selected glomeruli per kidney were evaluated.

#### Heart

Histological evaluation of the heart pathology was performed by light microscopy on H&E, PAS and Masson trichrome stained slides at 20X and 40X magnifications. At least two serial sections per mouse were evaluated in three non- pregnant A1Mko, four pregnant A1Mko at E12.5–13.5 and six pregnant A1Mko at E17.5, four non-pregnant WT, four pregnant WT at E12.5–13.5 and six pregnant WT at E17.5. The total amount of cardiac myocytes was counted on photographs acquired on standardized camera setting and 40X magnification (adopted with modification^69,70^). The number of cells within an 86 µm diameter circle in the tissue sections was counted in two randomly selected areas of the left compartment sectioned in a transverse plane for each heart. The amount of cell per µm^2^ was calculated as total amount of cells/A, where $A=\pi {r}^{2}$ and r = 43 µm.

#### Placenta

The placental width, the width of the labyrinth and junctional zones were measured three times on two sections from each placenta from the fetal surface near the umbilical cord insertion to the decidua, on H&E stained slides at 20X magnification.

Placental evaluation by TEM was performed on two A1Mko and two WT placentas at E13.5 and two A1Mko and two WT placentas at E17.5.

### Urine albumin and creatinine measurements

Urine albumin and creatinine levels were determined using Albuwell M and the Creatinine Companion commercial ELISA kits (Exocell, Philadelphia, PA, USA). In brief, stored urine samples were thawed and centrifuged for 30 s to pellet any cell particles. Supernatants were diluted with NBEBSA diluent or distilled water and assayed in accordance with the manufacturer’s standard protocol. All samples were run in duplicate with appropriate controls and blanks. The absorbance was read at 450 nm for albumin and 500 nm for creatinine. The absorbance obtained were fitted into a standard curve (albumin) or standard regression line (creatinine). Final calculations were performed with antilog 10 conversion (albumin) and delta calculations (creatinine) multiplied by the dilution factor, according to the manufacturer’s instructions.

### Blood urea nitrogen measurements

Blood urea nitrogen was measured by a quantitative colorimetric method using a QuantiChrom Urea Assay kit (BioAssay Systems, CA, USA). Briefly, stored plasma samples were thawed, vortexed and assayed directly as stipulated in the manufacturer’s protocol. All samples were run in duplicate with appropriate standards and blanks. The plate was read at 490 nm. The BUN concentration was calculated as a fraction of the sample absorbance (minus the blank) as the numerator and the absorbance of the standard (minus the blank) as the denominator, multiplied by the dilution factor.

### Gene profiling

Gene expression was determined with the commercially available real time RT^2^ Profiler PCR Array kits (Qiagen, CA, USA).

RNA extracts of kidney from three A1Mko and three WT females at E12.5–13.5 and kidneys and heart from five A1Mko and five WT females at E17.5 were assessed using the Mouse Hypertension array (Qiagen, catalog number PAMM 037Z). The Mouse Oxidative stress and Antioxidant Response array (Qiagen, catalog number PAMM 065Z) was used to analyze the placentas from three A1Mko and three WT females at E12.5–13.5 and the placentas and kidneys from five A1Mko and five WT females at E17.5.

All arrays were run according to the manufacturer’s protocol. In brief, all purified RNA samples from a particular group (A1Mko) or controls (WT) were pooled together by origin (kidney, heart or placenta) and gestational age (E12.5–13.5 or E17.5). Each pool was reverse transcribed with a cDNA synthesis kit. The synthesized cDNA was combined with RT^2^ SYBR Green qPCR Mastermix (Qiagen, catalog number 330529) and the qPCR was performed in a Bio-Rad CFX Connect Real-time System cycler (Bio-Rad Technologies, Denmark).

### Gene expression analysis for validation

Reverse transcription was done using RT^2^ First Strand Kit for cDNA synthesis (Qiagen, CA, USA). Real-time PCR was performed for *NPPB, MB, CPS1*, *MYLK2*, *APOE*, with Heat shock protein 90 alpha class B member 1 (*HSP90AB1*) as a house-keeping gene and using a Bio-Rad CFX Connect thermocycler (Bio-Rad Technologies, Denmark). The RT^2^ qPCR Primer Assays (Qiagen) specific for mouse *NPPB* (PPM04558A-200), *MB* (PPM05326C-200), *CPS1* (PPM41727A-200), *MYLK2* (PPM35075A-200), *APOE* (PPM04128B-200) and *HSP90AB1* (PPM04803F-200) was used together with RT^2^ SYBR Green Mastermix (Qiagen, catalog number 330529) according to manufacturer’s instructions. All samples were run in duplicates.

### Data analysis

Statistical analysis was performed with SPSS 26 software package (IBM, New York, USA), except for mixed regression modeling for repeated measurements, which was performed with the SAS Enterprise Guide (version 8.1, SAS Institute, Cary, NC, USA). Graphs for the means of the placental and fetal weights were created with the GraphPad Prism software (Version 8.04, San Diego, CA, USA).

All continuous variables for gross and microscopic evaluation of the organs are presented as median with minimum and maximum. Baseline values for SBP and HR were calculated as means of at least two weeks of measurement prior to conception. Mixed regression modeling was used for SBP and HR baseline and intragroup trend analysis.

Mann–Whitney test was used to compare continuous variables, with regards to the effect of genotype to the changes observed. Fisher’s exact test was used for analysis of effect of genotype for light pathological findings in the kidneys. P < 0.05 was considered statistically significant.

Gene expression analysis was performed using the Qiagen Data Analysis Web Portal (http//:www.giagen.com/geneglobe). Gene expression was calculated as fold change by the ΔΔ (delta delta) threshold cycle (C_T_) method and represented as an exponent of 2. A fold change of plus (+) or minus (−) 2 or higher corresponds to p < 0.05 by the Student’s T-test.

The validation of gene expression was done using single probes and the ΔΔCt method^71^. The p-values were calculated using Mann–Whitney test for each gene in the A1Mko and WT groups.

## Supplementary information

Supplementary Information.

## Data Availability

Materials, data and associated protocols are available on request.

## Abbreviations

A1M: Alpha-_1_-microglobulin
A1Mko: Alpha-_1_-microglobulin knockout
bpm: Beats per minute
BUN: Blood urea nitrogen
cDNA: Complementary DNA
E: Gestational day counting from visual copulation plug (E0.5)
H&E: Hematoxylin and eosin staining
HR: Heart rate
IUGR: Intrauterine growth restriction
mmHg: Millimeter mercury
n.s.: Not significant
PAS: Periodic acid Schiff staining
SBP: Systolic blood pressure
TEM: Transmission electron microscopy
WT: Wild type

## References

[1] Åkerström B, Gram M (2014). A1M, an extravascular tissue cleaning and housekeeping protein. Free Radic. Biol. Med..

[2] Tejler L, Eriksson S, Grubb A, Astedt B (1978). Production of protein HC by human fetal liver explants. Biochim. Biophys. Acta.

[3] Bouic P (1985). Alpha 1-microglobulin: a new antigenic component of the epidermo-dermal junction in normal human skin. Br. J. Dermatol..

[4] Berggard T (1999). Histologic distribution and biochemical properties of alpha 1-microglobulin in human placenta. Am. J. Reprod. Immunol..

[5] Allhorn M, Berggard T, Nordberg J, Olsson ML, Akerstrom B (2002). Processing of the lipocalin alpha(1)-microglobulin by hemoglobin induces heme-binding and heme-degradation properties. Blood.

[6] Allhorn M, Klapyta A, Akerstrom B (2005). Redox properties of the lipocalin alpha1-microglobulin: reduction of cytochrome c, hemoglobin, and free iron. Free Radic. Biol. Med..

[7] Åkerström B, Maghzal GJ, Winterbourn CC, Kettle AJ (2007). The lipocalin - has radical scavenging activity. J. Biol. Chem..

[8] Olsson MG (2013). The radical-binding lipocalin A1M binds to a Complex I subunit and protects mitochondrial structure and function. Antioxid. Redox Signal..

[9] Olsson MG (2012). Pathological conditions involving extracellular hemoglobin: molecular mechanisms, clinical significance, and novel therapeutic opportunities for alpha (1)-microglobulin. Antioxid. Redox Signal..

[10] Ahlstedt J, Tran TA, Strand SE, Gram M, Akerstrom B (2015). Human anti-oxidation protein A1M—a potential kidney protection agent in peptide receptor radionuclide therapy. Int. J. Mol. Sci..

[11] Wester-Rosenlof L (2014). A1M/alpha1-microglobulin protects from heme-induced placental and renal damage in a pregnant sheep model of preeclampsia. PLoS ONE.

[12] Naav A (2015). A1M ameliorates preeclampsia-like symptoms in placenta and kidney induced by cell-free fetal hemoglobin in rabbit. PLoS ONE.

[13] May K (2011). Perfusion of human placenta with hemoglobin introduces preeclampsia-like injuries that are prevented by alpha1-microglobulin. Placenta.

[14] Sverrisson K (2014). Extracellular fetal hemoglobin induces increases in glomerular permeability: inhibition with alpha-1-microglobulin and tempol. Am. J. Physiol. Renal Physiol..

[15] Olsson MG, Olofsson T, Tapper H, Åkerström B (2008). The lipocalin alpha1-microglobulin protects erythroid K562 cells against oxidative damage induced by heme and reactive oxygen species. Free Radic. Res..

[16] Olsson MG, Allhorn M, Olofsson T, Åkerström B (2007). Up-regulation of alpha1-microglobulin by hemoglobin and reactive oxygen species in hepatoma and blood cell lines. Free Radic. Biol. Med..

[17] Olsson MG (2011). Up-regulation of A1M/alpha1-microglobulin in skin by heme and reactive oxygen species gives protection from oxidative damage. PLoS ONE.

[18] Abalos E, Cuesta C, Grosso AL, Chou D, Say L (2013). Global and regional estimates of preeclampsia and eclampsia: a systematic review. Eur. J. Obstet. Gynecol. Reprod. Biol..

[19] Sutton ALM, Harper LM, Tita ATN (2018). Hypertensive disorders in pregnancy. Obstet. Gynecol. Clin. North Am..

[20] Redman CW, Sargent IL (2009). Placental stress and pre-eclampsia: a revised view. Placenta.

[21] Redman CW, Sargent IL, Staff AC (2014). IFPA Senior Award Lecture: making sense of pre-eclampsia—two placental causes of preeclampsia?. Placenta.

[22] Burton GJ, Jauniaux E (2004). Placental oxidative stress: from miscarriage to preeclampsia. J. Soc. Gynecol. Investig..

[23] Aouache R, Biquard L, Vaiman D, Miralles F (2018). Oxidative stress in preeclampsia and placental diseases. Int. J. Mol. Sci..

[24] Gram M (2015). The human endogenous protection system against cell-free hemoglobin and heme is overwhelmed in preeclampsia and provides potential biomarkers and clinical indicators. PLoS ONE.

[25] Kalapotharakos G (2019). Plasma Heme Scavengers Alpha-1-Microglobulin and Hemopexin as Biomarkers in High-Risk Pregnancies. Front. Physiol..

[26] Erlandsson L (2019). Alpha-1 microglobulin as a potential therapeutic candidate for treatment of hypertension and oxidative stress in the STOX1 preeclampsia mouse model. Sci. Rep..

[27] Bergwik J (2020). Knockout of the radical scavenger alpha1-microglobulin in mice results in defective bikunin synthesis, endoplasmic reticulum stress and increased body weight. Free Radic. Biol. Med..

[28] Pries AR, Secomb TW, Gaehtgens P (1995). Design principles of vascular beds. Circ. Res..

[29] West CA, Sasser JM, Baylis C (2016). The enigma of continual plasma volume expansion in pregnancy: critical role of the renin-angiotensin-aldosterone system. Am. J. Physiol. Renal Physiol..

[30] Thornburg KL, Jacobson SL, Giraud GD, Morton MJ (2000). Hemodynamic changes in pregnancy. Semin. Perinatol..

[31] Ogge G (2011). Placental lesions associated with maternal underperfusion are more frequent in early-onset than in late-onset preeclampsia. J. Perinat. Med..

[32] Goulopoulou S (2017). Maternal vascular physiology in preeclampsia. Hypertension.

[33] Carlstrom M, Wilcox CS, Arendshorst WJ (2015). Renal autoregulation in health and disease. Physiol. Rev..

[34] Brooks VL, Cassaglia PA, Zhao D, Goldman RK (2012). Baroreflex function in females: changes with the reproductive cycle and pregnancy. Gend. Med..

[35] Codsi E (2017). Longitudinal characterization of renal proximal tubular markers in normotensive and preeclamptic pregnancies. Am. J. Physiol. Regul. Integr. Comp. Physiol..

[36] Strevens H (2003). Glomerular endotheliosis in normal pregnancy and pre-eclampsia. BJOG.

[37] Cornelis T, Odutayo A, Keunen J, Hladunewich M (2011). The kidney in normal pregnancy and preeclampsia. Semin. Nephrol..

[38] Phipps E, Prasanna D, Brima W, Jim B (2016). Preeclampsia: updates in pathogenesis, definitions, and guidelines. Clin. J. Am. Soc. Nephrol..

[39] Moghaddas Sani H, Zununi Vahed S, Ardalan M (2019). Preeclampsia: a close look at renal dysfunction. Biomed. Pharmacother..

[40] Karumanchi SA, Maynard SE, Stillman IE, Epstein FH, Sukhatme VP (2005). Preeclampsia: a renal perspective. Kidney Int..

[41] Pollak VE, Nettles JB (1960). Preliminary observations on the differential diagnosis of toxemias of pregnancy by means of renal biopsy. Am. J. Obstet. Gynecol..

[42] Galvis-Ramirez MF, Quintana-Castillo JC, Bueno-Sanchez JC (2018). Novel insights into the role of glycans in the pathophysiology of glomerular endotheliosis in preeclampsia. Front. Physiol..

[43] Kawasoe S (2017). Mechanism of the blood pressure-lowering effect of sodium-glucose cotransporter 2 inhibitors in obese patients with type 2 diabetes. BMC. Pharmacol. Toxicol..

[44] Tsai EJ, Kass DA (2009). Cyclic GMP signaling in cardiovascular pathophysiology and therapeutics. Pharmacol. Ther..

[45] de Bold AJ (2001). The physiological and pathophysiological modulation of the endocrine function of the heart. Can. J. Physiol. Pharmacol..

[46] Davis MJ, Hill MA (1999). Signaling mechanisms underlying the vascular myogenic response. Physiol. Rev..

[47] Koshimizu TA (2012). Vasopressin V1a and V1b receptors: from molecules to physiological systems. Physiol. Rev..

[48] Evers KS, Wellmann S (2016). Arginine vasopressin and copeptin in perinatology. Front. Pediatr..

[49] Sandgren JA (2018). Arginine vasopressin infusion is sufficient to model clinical features of preeclampsia in mice. JCI Insight..

[50] Totzeck M (2012). Nitrite regulates hypoxic vasodilation via myoglobin-dependent nitric oxide generation. Circulation.

[51] Conrad KP, Gandley RE, Ogawa T, Nakanishi S, Danielson LA (1999). Endothelin mediates renal vasodilation and hyperfiltration during pregnancy in chronically instrumented conscious rats. Am. J. Physiol..

[52] Molnar M, Hertelendy F (1992). N omega-nitro-L-arginine, an inhibitor of nitric oxide synthesis, increases blood pressure in rats and reverses the pregnancy-induced refractoriness to vasopressor agents. Am. J. Obstet. Gynecol..

[53] Shiva S (2007). Nitrite augments tolerance to ischemia/reperfusion injury via the modulation of mitochondrial electron transfer. J. Exp. Med..

[54] Rutardottir S (2016). Structural and biochemical characterization of two heme binding sites on alpha1-microglobulin using site directed mutagenesis and molecular simulation. Biochim. Biophys. Acta.

[55] Tamura N (2000). Cardiac fibrosis in mice lacking brain natriuretic peptide. Proc. Natl. Acad. Sci. USA.

[56] Leung YK, Du J, Huang Y, Yao X (2010). Cyclic nucleotide-gated channels contribute to thromboxane A2-induced contraction of rat small mesenteric arteries. PLoS ONE.

[57] Szabo G, Molvarec A, Nagy B, Rigo J (2014). Increased B-type natriuretic peptide levels in early-onset versus late-onset preeclampsia. Clin. Chem. Lab. Med..

[58] Junus K, Wikstrom AK, Larsson A, Olovsson M (2017). Early second-trimester plasma levels of NT-proBNP in women who subsequently develop early-onset preeclampsia. J. Matern. Fetal. Neonatal Med..

[59] Ojeda NB, Grigore D, Alexander BT (2008). Intrauterine growth restriction: fetal programming of hypertension and kidney disease. Adv. Chronic Kidney Dis..

[60] Bujold E (2010). Prevention of preeclampsia and intrauterine growth restriction with aspirin started in early pregnancy: a meta-analysis. Obstet. Gynecol..

[61] Redman CW (1984). Maternal plasma volume and disorders of pregnancy. Br. Med. J..

[62] Majed BH, Khalil RA (2012). Molecular mechanisms regulating the vascular prostacyclin pathways and their adaptation during pregnancy and in the newborn. Pharmacol. Rev..

[63] Granger JP, Alexander BT, Llinas MT, Bennett WA, Khalil RA (2002). Pathophysiology of preeclampsia: linking placental ischemia/hypoxia with microvascular dysfunction. Microcirculation.

[64] Verlohren S (2008). Uterine vascular function in a transgenic preeclampsia rat model. Hypertension.

[65] Feng M (2008). Validation of volume-pressure recording tail-cuff blood pressure measurements. Am. J. Hypertens..

[66] Feng M, DiPetrillo K (2009). Non-invasive blood pressure measurement in mice. Methods Mol. Biol..

[67] Daugherty A, Rateri D, Hong L, Balakrishnan A (2009). Measuring blood pressure in mice using volume pressure recording, a tail-cuff method. J. Vis. Exp..

[68] Carlemalm E (1990). Lowicryl resins in microbiology. J. Struct. Biol..

[69] Bell ET (1936). The early stages of glomerulonephritis. Am. J. Path..

[70] Basgen JM, Rozen S, Nicholas S (2005). Comparison of methods for counting cells in tissue sections. Microsc. Microanal..

[71] Livak KJ, Schmittgen TD (2001). Analysis of relative gene expression data using real-time quantitative PCR and the 2(-Delta Delta C(T)) Method. Methods.

