# Supplementary material for: Pregnant alpha-1-microglobulin (A1M) knockout mice exhibit features of kidney and placental damage, hemodynamic changes and intrauterine growth restriction
Source: Sci Rep. 2020 Nov 26;10:20625. doi: 10.1038/s41598-020-77561-6 (PMC7691512; doi:10.1038/s41598-020-77561-6)
Supplement: Supplementary file 1 — Supplementary Information. [file 41598_2020_77561_MOESM1_ESM.docx]

**Pregnant alpha-1-microglobulin (A1M) knockout mice exhibit features of kidney and placental damage, hemodynamic changes and fetal growth restriction.**

**Larysa Aleksenko^1*^, Bo** **Åkerström^2^, Eva Hansson^1^, Lena Erlandsson^1^, Stefan R. Hansson^1,3^**

^1^Division of Obstetrics and Gynecology, Department of Clinical Sciences Lund, Lund University, Lund, Sweden

^2^Division of Infection Medicine, Department of Clinical Sciences Lund, Lund University, Lund, Sweden

^3^Skåne University Hospital, Obstetrics and Gynecology, Sweden

***Correspondence:**Larysa Aleksenko
larysa.aleksenko@med.lu.se

**Table S1.** Microscopic evaluation of the hearts, data is presented as median (minimum- maximum).

| **Parameters** | **A1Mko** | | | **WT** | | |
| --- | --- | --- | --- | --- | --- | --- |
|  | **Non**  **pregnant**  **(n=3)** | **E12.5-13.5 (n=4)** | **E17.5**  **(n=6)** | **Non pregnant**  **(n=4)** | **E12.5-13.5**  **(n=4)** | **E17.5**  **(n=6)** |
| Cell number per µm^2^ of tissue x 10^3^ | 8^a^  (7-9) | 9^b^  (6-11) | 8^c^  (5-10) | 7  (7- 10) | 8  (5-10) | 8  (6-10) |

Mann-Whitney test: ^a^ P=0.772, ^b^ P=0.564, ^c^ P=0.775

**Table S2.** Biochemical parameters in urine and blood, data is presented as median (minimum- maximum).

| **Parameters** | **A1Mko** | | | **WT** | | |
| --- | --- | --- | --- | --- | --- | --- |
|  | **Non**  **pregnant**  **(n=2)** | **E12.5-13.5 (n=4)** | **E17.5**  **(n=7)** | **Non pregnant**  **(n=2)** | **E12.5-13.5**  **(n=3)** | **E17.5**  **(n=3)** |
| Albumin urine  (mg/mL) | 3.4  (2.0-4.7) | 6.4  (1.4-25.0) | 4.8  (0.3-21.6) | 5.7  (2.9-8.5) | 14.8  (12.1-17.4) | 4.0  (2.3-14.2) |
| Creatinine urine  (mg/mL) | 2.7  (1.0-4.3) | 5.6  (1.9-11.6) | 2.6  (0.8-5.72) | 1.9  (1.8-2.0) | 5.1  (3.15-5.84) | 3.3  (1.8-10.1) |
| BUN (mg/mL) | 61.5  (58.7-64.3) | 59.2  (42.5-64.5) | 55.4  (27.6-69.0) | 59.4  (47.7-74.7) | 52.5  (41.3-56.7) | 53.5  (51.8-59.0) |

**Table S3**: Complete list of gene expression on the Mouse Hypertension and Mouse Oxidative stress and Antioxidant response RT^2^ PCR profiler arrays in A1Mko at E12.5-13.5 and E17.5

|  |  | **A1Mko** | |
| --- | --- | --- | --- |
|  |  | **E12.5-13.5** | **E17.5** |
| **RT2 Profiler PCR Array Mouse hypertension** | | | |
| **Kidney** | | | |
| *ACE* | Angiotensin I converting enzyme (peptidyl-dipeptidase A) 1 | -1.09 | -1.18 |
| *ACE2* | Angiotensin I converting enzyme (peptidyl-dipeptidase A) 2 | +1.53 | -1.32 |
| *ACTA2* | Actin, alpha 2, smooth muscle, aorta | +1.07 | +1.25 |
| *ADM* | Adrenomedullin | +1.12 | +1.09 |
| *ADRA1B* | Adrenergic receptor, alpha 1b | -1.17 | +1.71 |
| *ADRA1D* | Adrenergic receptor, alpha 1d | -1.11 | +2.67 |
| *ADRB1* | Adrenergic receptor, beta 1 | +1.10 | +1.76 |
| *AGT* | Angiotensinogen (serpin peptidase inhibitor, clade A, member 8) | -1.57 | +1.29 |
| *AGTR1A* | Angiotensin II receptor, type 1a | +1.15 | +1.19 |
| *AGTR1B* | Angiotensin II receptor, type 1b | +1.01 | -1.38 |
| *AGTR2* | Angiotensin II receptor, type 2 | -1.49 | -3.03 |
| *ALOX5* | Arachidonate 5-lipoxygenase | -1.06 | -1.23 |
| *ARG2* | Arginase type II | +1.60 | -1.13 |
| *ATP2C1* | ATPase, Ca++-sequestering | +1.07 | +1.03 |
| *ATP6AP2* | ATPase, H+ transporting, lysosomal accessory protein 2 | +1.61 | +1.08 |
| *AVP* | Arginine vasopressin | -1.03 | -1.08 |
| *AVPR1A* | Arginine vasopressin receptor 1A | -1.14 | +1.19 |
| *AVPR1B* | Arginine vasopressin receptor 1B | +1.14 | +3.35 |
| *BDKRB1* | Bradykinin receptor, beta 1 | -1.64 | -1.13 |
| *BDKRB2* | Bradykinin receptor, beta 2 | -1.49 | +2.21 |
| *BMPR2* | Bone morphogenic protein receptor, type II (serine/threonine kinase) | +1.44 | +1.03 |
| *CACNA1C* | Calcium channel, voltage-dependent, L type, alpha 1C subunit | -1.61 | +1.16 |
| *CALCA* | Calcitonin/calcitonin-related polypeptide, alpha | +1.10 | +1.26 |
| *CAV1* | Caveolin 1, caveolae protein | +1.68 | -1.09 |
| *CHRNA1* | Cholinergic receptor, nicotinic, alpha polypeptide 1 (muscle) | -1.03 | +1.10 |
| *CHRNB1* | Cholinergic receptor, nicotinic, beta polypeptide 1 (muscle) | -1.10 | +1.05 |
| *CLIC1* | Chloride intracellular channel 1 | +1.39 | +1.16 |
| *CLIC4* | Chloride intracellular channel 4 (mitochondrial) | +1.02 | +1.26 |
| *CLIC5* | Chloride intracellular channel 5 | +1.00 | +1.11 |
| *CNGA1* | Cyclic nucleotide gated channel alpha 1 | +1.20 | +2.07 |
| *CNGA2* | Cyclic nucleotide gated channel alpha 2 | -1.19 | +3.12 |
| *CNGA3* | Cyclic nucleotide gated channel alpha 3 | -1.51 | +2.45 |
| *CNGA4* | Cyclic nucleotide gated channel alpha 4 | +1.52 | +1.13 |
| *CNGB1* | Cyclic nucleotide gated channel beta 1 | +1.05 | +2.17 |
| *CNGB3* | Cyclic nucleotide gated channel beta 3 | -1.03 | -1.08 |
| *CPS1* | Carbamoyl-phosphate synthetase 1 | -1.60 | -2.39 |
| *DRD3* | Dopamine receptor D3 | -2.12 | +1.43 |
| *DRD5* | Dopamine receptor D5 | -1.03 | -1.08 |
| *ECE1* | Endothelin converting enzyme 1 | -1.17 | +1.15 |
| *EDN1* | Endothelin 1 | +1.23 | +1.15 |
| *EDN2* | Endothelin 2 | -1.29 | +2.71 |
| *EDNRA* | Endothelin receptor type A | +1.10 | +1.47 |
| *EDNRB* | Endothelin receptor type B | +1.25 | +1.12 |
| *EPHX2* | Epoxide hydrolase 2, cytoplasmic | +1.00 | +1.40 |
| *GCH1* | GTP cyclohydrolase 1 | +1.06 | -1.44 |
| *GCHFR* | GTP cyclohydrolase I feedback regulator | +1.79 | +1.37 |
| *GUCY1A3* | Guanylate cyclase 1, soluble, alpha 3 | +1.44 | +1.40 |
| *GUCY1B3* | Guanylate cyclase 1, soluble, beta 3 | +1.31 | +1.03 |
| *HIF1A* | Hypoxia inducible factor 1, alpha subunit | +1.46 | -1.02 |
| *ITPR1* | Inositol 1,4,5-trisphosphate receptor 1 | +1.02 | +1.23 |
| *ITPR2* | Inositol 1,4,5-triphosphate receptor 2 | +1.07 | +1.16 |
| *KCNJ8* | Potassium inwardly-rectifying channel, subfamily J, member 8 | +1.64 | +1.26 |
| *KCNMA1* | Potassium large conductance calcium-activated channel, subfamily M, alpha member 1 | +1.06 | +1.13 |
| *KNG1* | Kininogen 1 | -1.55 | -2.01 |
| *MYLK* | Myosin, light polypeptide kinase | +1.13 | +1.26 |
| *MYLK2* | Myosin, light polypeptide kinase 2, skeletal muscle | -1.21 | +4.22 |
| *NOS3* | Nitric oxide synthase 3, endothelial cell | -1.12 | +1.72 |
| *NOSIP* | Nitric oxide synthase interacting protein | +1.61 | -1.04 |
| *NOSTRIN* | Nitric oxide synthase trafficker | +1.86 | +1.41 |
| *NPPB* | Natriuretic peptide type B | +13.16 | -1.08 |
| *NPPC* | Natriuretic peptide type C | +1.24 | +1.60 |
| *NPR1* | Natriuretic peptide receptor 1 | -1.62 | +1.18 |
| *NPY1R* | Neuropeptide Y receptor Y1 | +1.19 | +1.27 |
| *P2RX4* | Purinergic receptor P2X, ligand-gated ion channel 4 | +1.14 | +1.15 |
| *PDE3A* | Phosphodiesterase 3A, cGMP inhibited | +1.22 | +1.29 |
| *PDE3B* | Phosphodiesterase 3B, cGMP-inhibited | +1.51 | +1.10 |
| *PDE5A* | Phosphodiesterase 5A, cGMP-specific | +1.21 | +1.27 |
| *PLCG1* | Phospholipase C, gamma 1 | -1.06 | -1.01 |
| *PLCG2* | Phospholipase C, gamma 2 | -1.25 | +1.18 |
| *PRKG1* | Protein kinase, cGMP-dependent, type I | +1.44 | +1.26 |
| *PRKG2* | Protein kinase, cGMP-dependent, type II | +1.01 | +1.72 |
| *PTGIR* | Prostaglandin I receptor (IP) | -1.31 | +2.25 |
| *PTGS1* | Prostaglandin-endoperoxide synthase 1 | +1.02 | +1.29 |
| *PTGS2* | Prostaglandin-endoperoxide synthase 2 | +1.01 | +2.07 |
| *REN1* | Renin 1 structural | -1.84 | +1.22 |
| *S1PR1* | Sphingosine-1-phosphate receptor 1 | -1.47 | +1.29 |
| *SCNN1A* | Sodium channel, nonvoltage-gated 1 alpha | -1.28 | +1.04 |
| *SCNN1B* | Sodium channel, nonvoltage-gated 1 beta | -1.11 | +1.35 |
| *SCNN1G* | Sodium channel, nonvoltage-gated 1 gamma | +1.36 | +1.23 |
| *SLC7A1* | Solute carrier family 7 (cationic amino acid transporter, y+ system), member 1 | -1.47 | +1.01 |
| *SPHK1* | Sphingosine kinase 1 | -1.34 | +1.13 |
| *SPHK2* | Sphingosine kinase 2 | +1.10 | +1.09 |
| *UTS2* | Urotensin 2 | -1.31 | +2.21 |
| *UTS2R* | Urotensin 2 receptor | -1.03 | +2.59 |
| **Heart** | | | |
| *ACE* | Angiotensin I converting enzyme (peptidyl-dipeptidase A) 1 | - | +1.13 |
| *ACE2* | Angiotensin I converting enzyme (peptidyl-dipeptidase A) 2 | - | +1.08 |
| *ACTA2* | Actin, alpha 2, smooth muscle, aorta | - | -1.21 |
| *ADM* | Adrenomedullin | - | -1.36 |
| *ADRA1B* | Adrenergic receptor, alpha 1b | - | +1.33 |
| *ADRA1D* | Adrenergic receptor, alpha 1d | - | +1.70 |
| *ADRB1* | Adrenergic receptor, beta 1 | - | +1.36 |
| *AGT* | Angiotensinogen (serpin peptidase inhibitor, clade A, member 8) | - | -1.03 |
| *AGTR1A* | Angiotensin II receptor, type 1a | - | +1.04 |
| *AGTR1B* | Angiotensin II receptor, type 1b | - | +1.22 |
| *AGTR2* | Angiotensin II receptor, type 2 | - | +2.59 |
| *ALOX5* | Arachidonate 5-lipoxygenase | - | +1.07 |
| *ARG2* | Arginase type II | - | +1.09 |
| *ATP2C1* | ATPase, Ca++-sequestering | - | +1.32 |
| *ATP6AP2* | ATPase, H+ transporting, lysosomal accessory protein 2 | - | +1.13 |
| *AVP* | Arginine vasopressin | - | +1.22 |
| *AVPR1A* | Arginine vasopressin receptor 1A | - | +1.25 |
| *AVPR1B* | Arginine vasopressin receptor 1B | - | +1.04 |
| *BDKRB1* | Bradykinin receptor, beta 1 | - | +1.22 |
| *BDKRB2* | Bradykinin receptor, beta 2 | - | +1.22 |
| *BMPR2* | Bone morphogenic protein receptor, type II (serine/threonine kinase) | - | +1.05 |
| *CACNA1C* | Calcium channel, voltage-dependent, L type, alpha 1C subunit | - | -1.17 |
| *CALCA* | Calcitonin/calcitonin-related polypeptide, alpha | - | +1.40 |
| *CAV1* | Caveolin 1, caveolae protein | - | +1.01 |
| *CHRNA1* | Cholinergic receptor, nicotinic, alpha polypeptide 1 (muscle) | - | +1.47 |
| *CHRNB1* | Cholinergic receptor, nicotinic, beta polypeptide 1 (muscle) | - | -1.07 |
| *CLIC1* | Chloride intracellular channel 1 | - | -1.00 |
| *CLIC4* | Chloride intracellular channel 4 (mitochondrial) | - | +1.17 |
| *CLIC5* | Chloride intracellular channel 5 | - | -1.08 |
| *CNGA1* | Cyclic nucleotide gated channel alpha 1 | - | +1.00 |
| *CNGA2* | Cyclic nucleotide gated channel alpha 2 | - | +1.37 |
| *CNGA3* | Cyclic nucleotide gated channel alpha 3 | - | +1.20 |
| *CNGA4* | Cyclic nucleotide gated channel alpha 4 | - | +1.15 |
| *CNGB1* | Cyclic nucleotide gated channel beta 1 | - | +1.16 |
| *CNGB3* | Cyclic nucleotide gated channel beta 3 | - | +1.22 |
| *CPS1* | Carbamoyl-phosphate synthetase 1 | - | -8.81 |
| *DRD3* | Dopamine receptor D3 | - | +1.43 |
| *DRD5* | Dopamine receptor D5 | - | +1.22 |
| *ECE1* | Endothelin converting enzyme 1 | - | +1.18 |
| *EDN1* | Endothelin 1 | - | +1.03 |
| *EDN2* | Endothelin 2 | - | +1.20 |
| *EDNRA* | Endothelin receptor type A | - | -1.08 |
| *EDNRB* | Endothelin receptor type B | - | +1.04 |
| *EPHX2* | Epoxide hydrolase 2, cytoplasmic | - | -1.04 |
| *GCH1* | GTP cyclohydrolase 1 | - | -1.01 |
| *GCHFR* | GTP cyclohydrolase I feedback regulator | - | +1.17 |
| *GUCY1A3* | Guanylate cyclase 1, soluble, alpha 3 | - | +1.27 |
| *GUCY1B3* | Guanylate cyclase 1, soluble, beta 3 | - | -1.03 |
| *HIF1A* | Hypoxia inducible factor 1, alpha subunit | - | +1.16 |
| *ITPR1* | Inositol 1,4,5-trisphosphate receptor 1 | - | +1.18 |
| *ITPR2* | Inositol 1,4,5-triphosphate receptor 2 | - | +1.02 |
| *KCNJ8* | Potassium inwardly-rectifying channel, subfamily J, member 8 | - | +1.07 |
| *KCNMA1* | Potassium large conductance calcium-activated channel, subfamily M, alpha member 1 | - | -1.34 |
| *KNG1* | Kininogen 1 | - | -18.94 |
| *MYLK* | Myosin, light polypeptide kinase | - | -1.13 |
| *MYLK2* | Myosin, light polypeptide kinase 2, skeletal muscle | - | +2.04 |
| *NOS3* | Nitric oxide synthase 3, endothelial cell | - | +1.08 |
| *NOSIP* | Nitric oxide synthase interacting protein | - | -1.01 |
| *NOSTRIN* | Nitric oxide synthase trafficker | - | -1.01 |
| *NPPB* | Natriuretic peptide type B | - | -1.06 |
| *NPPC* | Natriuretic peptide type C | - | +1.04 |
| *NPR1* | Natriuretic peptide receptor 1 | - | +1.02 |
| *NPY1R* | Neuropeptide Y receptor Y1 | - | -1.17 |
| *P2RX4* | Purinergic receptor P2X, ligand-gated ion channel 4 | - | +1.08 |
| *PDE3A* | Phosphodiesterase 3A, cGMP inhibited | - | +1.11 |
| *PDE3B* | Phosphodiesterase 3B, cGMP-inhibited | - | +1.30 |
| *PDE5A* | Phosphodiesterase 5A, cGMP-specific | - | -1.07 |
| *PLCG1* | Phospholipase C, gamma 1 | - | +1.16 |
| *PLCG2* | Phospholipase C, gamma 2 | - | -1.18 |
| *PRKG1* | Protein kinase, cGMP-dependent, type I | - | -1.03 |
| *PRKG2* | Protein kinase, cGMP-dependent, type II | - | +1.35 |
| *PTGIR* | Prostaglandin I receptor (IP) | - | +1.17 |
| *PTGS1* | Prostaglandin-endoperoxide synthase 1 | - | -1.21 |
| *PTGS2* | Prostaglandin-endoperoxide synthase 2 | - | -1.07 |
| *REN1* | Renin 1 structural | - | +1.14 |
| *S1PR1* | Sphingosine-1-phosphate receptor 1 | - | -1.08 |
| *SCNN1A* | Sodium channel, nonvoltage-gated 1 alpha | - | +1.11 |
| *SCNN1B* | Sodium channel, nonvoltage-gated 1 beta | - | +1.22 |
| *SCNN1G* | Sodium channel, nonvoltage-gated 1 gamma | - | +1.12 |
| *SLC7A1* | Solute carrier family 7 (cationic amino acid transporter, y+ system), member 1 | - | +1.05 |
| *SPHK1* | Sphingosine kinase 1 | - | +1.37 |
| *SPHK2* | Sphingosine kinase 2 | - | +1.30 |
| *UTS2* | Urotensin 2 | - | -1.39 |
| *UTS2R* | Urotensin 2 receptor | - | +1.68 |
| **RT2 Profiler PCR Array Oxidative Stress and Antioxidant Response** | | | |
| **Placenta** | | | |
| *ALS2* | Amyotrophic lateral sclerosis 2 (juvenile) homolog (human) | +1.12 | -1.02 |
| *AOX1* | Aldehyde oxidase 1 | -1.36 | -2.15 |
| *APC* | Adenomatosis polyposis coli | -1.21 | -1.06 |
| *APOE* | Apolipoprotein E | -3.17 | -1.21 |
| *ATR* | Ataxia telangiectasia and rad3 related | +1.03 | -1.06 |
| *CAT* | Catalase | -1.14 | -1.30 |
| *CCL5* | Chemokine (C-C motif) ligand 5 | -2.31 | +1.50 |
| *CCS* | Copper chaperone for superoxide dismutase | -1.39 | -1.10 |
| *CTSB* | Cathepsin B | +1.30 | +1.13 |
| *CYBA* | Cytochrome b-245, alpha polypeptide | -1.44 | -1.39 |
| *CYGB* | Cytoglobin | -1.28 | -1.56 |
| *DNM2* | Dynamin 2 | +1.15 | +1.00 |
| *DUOX1* | Dual oxidase 1 | -1.01 | -1.36 |
| *EHD2* | EH-domain containing 2 | -3.07 | +1.12 |
| *EPX* | Eosinophil peroxidase | +1.09 | +1.05 |
| *ERCC2* | Excision repair cross-complementing rodent repair deficiency, complementation group 2 | -1.15 | -1.23 |
| *ERCC6* | Excision repair cross-complementing rodent repair deficiency, complementation group 6 | -1.01 | -1.27 |
| *FANCC* | Fanconi anemia, complementation group C | +1.21 | -1.05 |
| *FMO2* | Flavin containing monooxygenase 2 | -3.29 | +1.25 |
| *FTH1* | Ferritin heavy chain 1 | +1.19 | -1.41 |
| *GCLC* | Glutamate-cysteine ligase, catalytic subunit | -1.15 | -1.32 |
| *GCLM* | Glutamate-cysteine ligase, modifier subunit | -1.11 | -1.20 |
| *GPX1* | Glutathione peroxidase 1 | +1.33 | -1.14 |
| *GPX2* | Glutathione peroxidase 2 | -2.94 | -1.14 |
| *GPX3* | Glutathione peroxidase 3 | -1.08 | -1.13 |
| *GPX4* | Glutathione peroxidase 4 | +1.56 | -1.04 |
| *GPX5* | Glutathione peroxidase 5 | +1.17 | -2.43 |
| *GPX6* | Glutathione peroxidase 6 | +1.15 | -1.15 |
| *GPX7* | Glutathione peroxidase 7 | -1.56 | -1.58 |
| *GSR* | Glutathione reductase | -1.85 | -1.04 |
| *GSS* | Glutathione synthetase | -1.09 | -1.40 |
| *GSTK1* | Glutathione S-transferase kappa 1 | +1.34 | -1.13 |
| *GSTP1* | Glutathione S-transferase, pi 1 | -1.23 | -1.15 |
| *HMOX1* | Heme oxygenase (decycling) 1 | +1.09 | +1.23 |
| *HSPALA* | Heat shock protein 1A | +1.14 | -1.54 |
| *IDH1* | Isocitrate dehydrogenase 1 (NADP+), soluble | -1.21 | -1.28 |
| *IFT172* | Intraflagellar transport 172 homolog (Chlamydomonas) | -1.14 | +1.02 |
| *IL19* | Interleukin 19 | +1.60 | -1.46 |
| *IL22* | Interleukin 22 | +1.15 | +1.05 |
| *KRT1* | Keratin 1 | -3.26 | -4.64 |
| *LPO* | Lactoperoxidase | +1.15 | +1.67 |
| *MB* | Myoglobin | +1.72 | +1.01 |
| *MPO* | Myeloperoxidase | -4.12 | +1.05 |
| *NCF1* | Neutrophil cytosolic factor 1 | -1.57 | +1.13 |
| *NCF2* | Neutrophil cytosolic factor 2 | -1.14 | -1.06 |
| *NGB* | Neuroglobin | +1.95 | +1.05 |
| *NOS2* | Nitric oxide synthase 2, inducible | -1.07 | +1.14 |
| *NOX1* | NADPH oxidase 1 | -1.44 | +1.05 |
| *NOX4* | NADPH oxidase 4 | -1.25 | -1.19 |
| *NOXA1* | NADPH oxidase activator 1 | +1.08 | -2.27 |
| *NOXO1* | NADPH oxidase organizer 1 | +1.60 | -1.62 |
| *NQO1* | NAD(P)H dehydrogenase, quinone 1 | -1.09 | +1.07 |
| *PARK7* | Parkinson disease (autosomal recessive, early onset) 7 | -1.05 | -1.16 |
| *PRDX1* | Peroxiredoxin 1 | +1.13 | -1.24 |
| *PRDX2* | Peroxiredoxin 2 | -1.79 | -1.13 |
| *PRDX3* | Peroxiredoxin 3 | -1.22 | -1.07 |
| *PRDX4* | Peroxiredoxin 4 | +1.84 | -1.17 |
| *PRDX5* | Peroxiredoxin 5 | +1.26 | -1.09 |
| *PRDX6* | Peroxiredoxin 6 | -1.62 | 1.00 |
| *PRNP* | Prion protein | -1.80 | -1.13 |
| *PSMB5* | Proteasome (prosome, macropain) subunit, beta type 5 | +1.22 | -1.14 |
| *PTGS1* | Prostaglandin-endoperoxide synthase 1 | -1.36 | -1.12 |
| *PTGS2* | Prostaglandin-endoperoxide synthase 2 | -1.29 | +1.14 |
| *RAG2* | Recombination activating gene 2 | +1.15 | -1.53 |
| *RECQ14* | RecQ protein-like 4 | -1.34 | -1.80 |
| *SCD1* | Stearoyl-Coenzyme A desaturase 1 | +1.03 | -1.11 |
| *SERPINB1B* | Serine (or cysteine) peptidase inhibitor, clade B, member 1b | -1.12 | -1.19 |
| *SLC38A1* | Solute carrier family 38, member 1 | +1.10 | -1.10 |
| *SOD1* | Superoxide dismutase 1, soluble | +1.19 | -1.18 |
| *SOD2* | Superoxide dismutase 2, mitochondrial | +1.10 | -1.18 |
| *SOD3* | Superoxide dismutase 3, extracellular | -1.46 | -1.80 |
| *SQSTM1* | Sequestosome 1 | +1.34 | -1.07 |
| *SRXN1* | Sulfiredoxin 1 homolog (S. cerevisiae) | +1.13 | +1.28 |
| *TPO* | Thyroid peroxidase | +1.15 | +1.05 |
| *TXN1* | Thioredoxin 1 | +1.71 | -1.69 |
| *TXNIP* | Thioredoxin interacting protein | -1.21 | -1.23 |
| *TXNRD1* | Thioredoxin reductase 1 | -1.17 | +1.01 |
| *TXNRD2* | Thioredoxin reductase 2 | +2.21 | -1.12 |
| *TXNRD3* | Thioredoxin reductase 3 | -1.48 | -1.08 |
| *UCP2* | Uncoupling protein 2 (mitochondrial, proton carrier) | -1.03 | +1.08 |
| *UCP3* | Uncoupling protein 3 (mitochondrial, proton carrier) | +1.15 | +1.12 |
| *VIM* | Vimentin | -1.57 | -1.01 |
| *XPA* | Xeroderma pigmentosum, complementation group A | +1.23 | -1.19 |
| **Kidney** |  |  |  |
| *ALS2* | Amyotrophic lateral sclerosis 2 (juvenile) homolog (human) | - | -1.11 |
| *AOX1* | Aldehyde oxidase 1 | - | -2.10 |
| *APC* | Adenomatosis polyposis coli | - | -1.01 |
| *APOE* | Apolipoprotein E | - | +1.17 |
| *ATR* | Ataxia telangiectasia and rad3 related | - | -1.03 |
| *CAT* | Catalase | - | +1.03 |
| *CCl5* | Chemokine (C-C motif) ligand 5 | - | +1.74 |
| *CCS* | Copper chaperone for superoxide dismutase | - | +1.13 |
| *CTSB* | Cathepsin B | - | -1.06 |
| *CYBA* | Cytochrome b-245, alpha polypeptide | - | -1.22 |
| *CYGB* | Cytoglobin | - | +1.08 |
| *DNM2* | Dynamin 2 | - | +1.03 |
| *DUOX1* | Dual oxidase 1 | - | +1.69 |
| *EHD2* | EH-domain containing 2 | - | +1.12 |
| *EPX* | Eosinophil peroxidase | - | +1.01 |
| *ERCC2* | Excision repair cross-complementing rodent repair deficiency, complementation group 2 | - | +1.02 |
| *ERCC6* | Excision repair cross-complementing rodent repair deficiency, complementation group 6 | - | +1.26 |
| *FANCC* | Fanconi anemia, complementation group C | - | +1.07 |
| *FMO2* | Flavin containing monooxygenase 2 | - | -1.06 |
| *FTH1* | Ferritin heavy chain 1 | - | -1.05 |
| *GCLC* | Glutamate-cysteine ligase, catalytic subunit | - | -1.15 |
| *GCLM* | Glutamate-cysteine ligase, modifier subunit | - | +1.06 |
| *GPX1* | Glutathione peroxidase 1 | - | -1.11 |
| *GPX2* | Glutathione peroxidase 2 | - | +1.01 |
| *GPX3* | Glutathione peroxidase 3 | - | -1.04 |
| *GPX4* | Glutathione peroxidase 4 | - | -1.21 |
| *GPX5* | Glutathione peroxidase 5 | - | +2.13 |
| *GPX6* | Glutathione peroxidase 6 | - | -1.02 |
| *GPX7* | Glutathione peroxidase 7 | - | -1.21 |
| *GSR* | Glutathione reductase | - | +1.12 |
| *GSS* | Glutathione synthetase | - | +1.03 |
| *GSTK1* | Glutathione S-transferase kappa 1 | - | -1.08 |
| *GSTP1* | Glutathione S-transferase, pi 1 | - | +1.04 |
| *HMOX1* | Heme oxygenase (decycling) 1 | - | -1.21 |
| *HSPALA* | Heat shock protein 1A | - | +1.09 |
| *IDH1* | Isocitrate dehydrogenase 1 (NADP+), soluble | - | -1.10 |
| *IFT172* | Intraflagellar transport 172 homolog (Chlamydomonas) | - | +1.26 |
| *IL19* | Interleukin 19 | - | +1.01 |
| *IL22* | Interleukin 22 | - | +1.01 |
| *KRT1* | Keratin 1 | - | +1.91 |
| *LPO* | Lactoperoxidase | - | +1.06 |
| *MB* | Myoglobin | - | -76.54 |
| *MPO* | Myeloperoxidase | - | +1.01 |
| *NCF1* | Neutrophil cytosolic factor 1 | - | +1.27 |
| *NCF2* | Neutrophil cytosolic factor 2 | - | +1.30 |
| *NGB* | Neuroglobin | - | +1.49 |
| *NOS2* | Nitric oxide synthase 2, inducible | - | +2.44 |
| *NOX1* | NADPH oxidase 1 | - | +1.52 |
| *NOX4* | NADPH oxidase 4 | - | +1.01 |
| *NOXA1* | NADPH oxidase activator 1 | - | +2.66 |
| *NOXO1* | NADPH oxidase organizer 1 | - | +1.89 |
| *NQO1* | NAD(P)H dehydrogenase, quinone 1 | - | -1.04 |
| *PARK7* | Parkinson disease (autosomal recessive, early onset) 7 | - | -1.04 |
| *PRDX1* | Peroxiredoxin 1 | - | -1.13 |
| *PRDX2* | Peroxiredoxin 2 | - | +1.06 |
| *PRDX3* | Peroxiredoxin 3 | - | +1.01 |
| *PRDX4* | Peroxiredoxin 4 | - | -1.14 |
| *PRDX5* | Peroxiredoxin 5 | - | +1.01 |
| *PRDX6* | Peroxiredoxin 6 | - | +1.04 |
| *PRNP* | Prion protein | - | +1.53 |
| *PSMB5* | Proteasome (prosome, macropain) subunit, beta type 5 | - | -1.01 |
| *PTGS1* | Prostaglandin-endoperoxide synthase 1 | - | +1.08 |
| *PTGS2* | Prostaglandin-endoperoxide synthase 2 | - | +2.06 |
| *RAG2* | Recombination activating gene 2 | - | +1.04 |
| *RECQ14* | RecQ protein-like 4 | - | +1.66 |
| *SCD1* | Stearoyl-Coenzyme A desaturase 1 | - | +1.49 |
| *SERPINB1B* | Serine (or cysteine) peptidase inhibitor, clade B, member 1b | - | -1.10 |
| *SLC38A1* | Solute carrier family 38, member 1 | - | +1.40 |
| *SOD1* | Superoxide dismutase 1, soluble | - | -1.14 |
| *SOD2* | Superoxide dismutase 2, mitochondrial | - | -1.10 |
| *SOD3* | Superoxide dismutase 3, extracellular | - | +1.15 |
| *SQSTM1* | Sequestosome 1 | - | -1.09 |
| *SRXN1* | Sulfiredoxin 1 homolog (S. cerevisiae) | - | -1.03 |
| *TPO* | Thyroid peroxidase | - | +1.01 |
| *TXN1* | Thioredoxin 1 | - | +1.21 |
| *TXNIP* | Thioredoxin interacting protein | - | +1.28 |
| *TXNRD1* | Thioredoxin reductase 1 | - | -1.06 |
| *TXNRD2* | Thioredoxin reductase 2 | - | +1.09 |
| *TXNRD3* | Thioredoxin reductase 3 | - | +1.36 |
| *UCP2* | Uncoupling protein 2 (mitochondrial, proton carrier) | - | -1.02 |
| *UCP3* | Uncoupling protein 3 (mitochondrial, proton carrier) | - | +1.01 |
| *VIM* | Vimentin | - | +1.08 |
| *XPA* | Xeroderma pigmentosum, complementation group A | - | +1.00 |

**
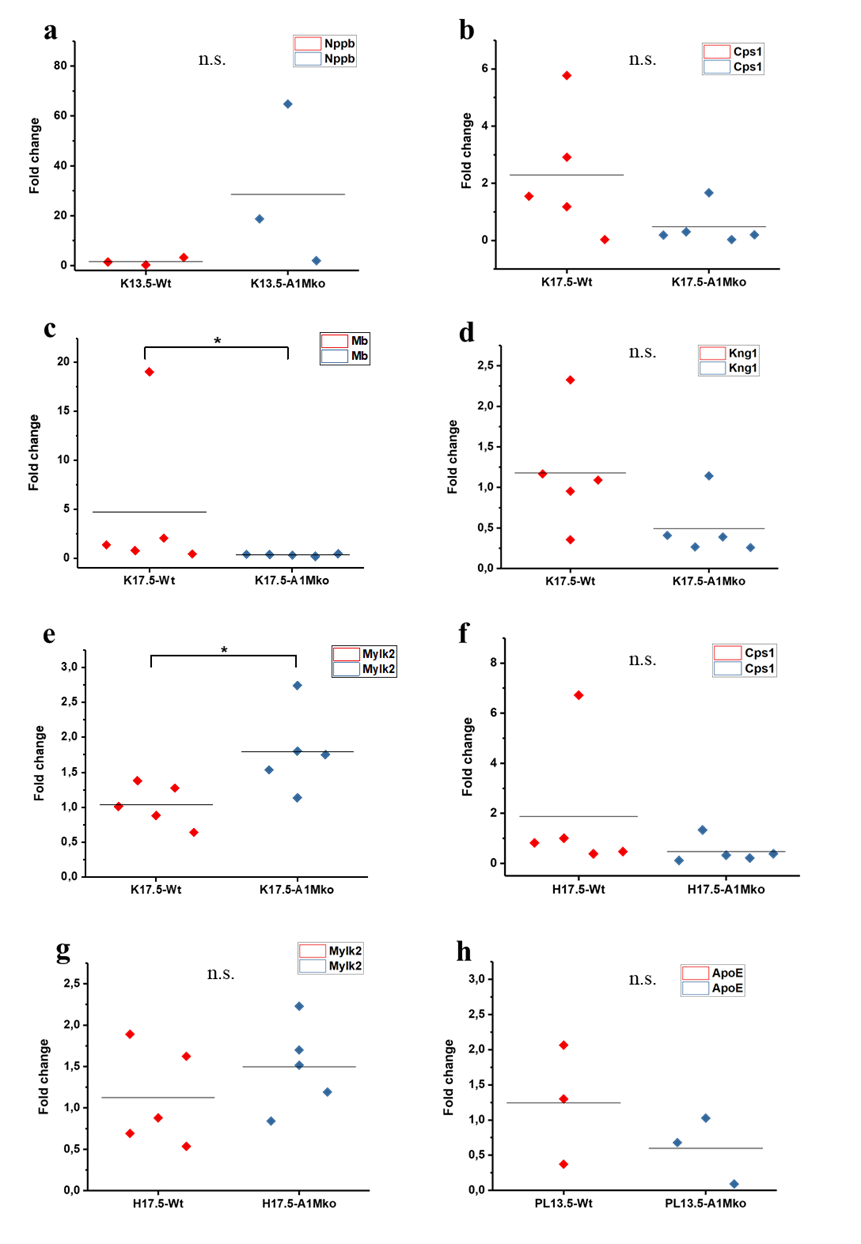
**

**Figure S1**. Real-time qPCR validation of genes NPPB, CPS1, MB, KNG1, MYLK2 and APOE. (**a**) NPPB expression in the kidney at E12.5-13.5. (**b**) CPS1 expression in the kidney at E17.5. (**c**) MB expression in the kidney at E17.5. (**d**) KNG1 expression in the kidney at E17.5. (**e**) MYLK2 expression in the kidney at E17.5. (**f**) CPS1 expression in the heart at E17.5. (**g**) MYLK2 expression in the heart at E17.5. (**h**) APOE expression in the placenta at E12.5-13.5. Mean is represented by the horizontal lines in each figure.
